# Transcriptome and DNA methylome reveal insights into yield heterosis in the curds of broccoli (*Brassica oleracea* L var. *italic*)

**DOI:** 10.1186/s12870-018-1384-4

**Published:** 2018-08-13

**Authors:** Hui Li, Jiye Yuan, Mei Wu, Zhanpin Han, Lihong Li, Hanmin Jiang, Yinglan Jia, Xue Han, Min Liu, Deling Sun, Chengbin Chen, Wenqin Song, Chunguo Wang

**Affiliations:** 1 College of Life Sciences, Nankai University, Tianjin, China; 2 College of Horticulture and Landscape, Tianjin Agricultural University, Tianjin, China; 3 Tianjin Kernel Vegetable Research Institute, Tianjin, China; 4 College of Life Sciences, Shandong Normal University, Jinan, Shandong China

**Keywords:** Broccoli (*Brassica oleracea* L var. *italic*), Curd, Heterosis, Transcriptome, DNA methylation

## Abstract

**Background:**

Curds are the main edible organs, which exhibit remarkable yield heterosis in F_1_ hybrid broccoli. However, the molecular basis underlying heterosis in broccoli remains elusive.

**Results:**

In the present study, transcriptome profiles revealed that the hybridization made most genes show additive expression patterns in hybrid broccoli. The differentially expressed genes including the non-additively expressed genes detected in the hybrid broccoli and its parents were mainly involved in light, hormone and hydrogen peroxide-mediated signaling pathways, responses to stresses, and regulation of floral development, which suggested that these biological processes should play crucial roles in the yield heterosis of broccoli. Among them, light and hydrogen peroxide-mediated signaling pathways represent two novel classes of regulatory processes that could function in yield or biomass heterosis of plants. Totally, 53 candidate genes closely involved in curd yield heterosis were identified. Methylome data indicated that the DNA methylation ratio of the hybrids was higher than that of their parents. However, the DNA methylation levels of most sites also displayed additive expression patterns. These sites with differential methylation levels were predominant in the intergenic regions. In most cases, the changes of DNA methylation levels in gene regions did not significantly affect their expression levels.

**Conclusions:**

The differentially expressed genes, the regulatory processes and the possible roles of DNA methylation modification in the formation of curd yield heterotic trait were discovered. These findings provided comprehensive insights into the curd yield heterosis in broccoli, and were significant for breeding high-yield broccoli varieties.

**Electronic supplementary material:**

The online version of this article (10.1186/s12870-018-1384-4) contains supplementary material, which is available to authorized users.

## Background

Heterosis or hybrid vigor refers to the phenomenon in which the F_1_ hybrids have higher biomass, yields, growth rates and resistance to environmental stresses than their parents [1, 2]. In agriculture, heterosis has been used extensively in breeding high yield corps. The plantations of hybrid rice, maize, sorghum, and wheat all worldwide have significantly contributed to increasing the grain yield and ensuring global food security [3]. Although heterosis has been successfully utilized in crop breeding practices, the molecular mechanism of heterosis remains poorly understood, and the explorations involved in how and why the F_1_ hybrids exhibit superior performance in plants never stopped since this phenomenon was discovered [4]. Previous studies have proposed three main genetic hypotheses, namely, dominance [5], over-dominance [6, 7] and epistasis [8], to explain the formation of heterosis in diverse plant species. The dominance hypothesis posited that the inferior parental alleles in the hybrids are complemented by the superior or dominant alleles from the other parent. The over-dominance hypothesis suggested that heterosis arises from the allelic interactions within each of many genetic loci. The epistasis hypothesis emphasized that the interactions between different parental genes in hybrids lead to heterosis. However, these three models were mainly theoretical, and none of them could fully explain the heterosis in plants. With the development of quantitative genetics, quantitative trait locus (QTL) mapping studies were widely conducted to identify the gene loci that controlled the heterotic traits. These works confirmed the involvement of many QTLs in biomass or yield heterosis in several crops, such as rice and maize [9–11], thereby providing potential molecular markers for further cloning the genes regulating this phenomenon. In recent years, transcriptome analysis based on high-throughput sequencing technologies offered a powerful strategy for exploring genome-wide gene expression profiles and confirming differentially expressed genes (DEGs) in hybrids and their parents, which may be directly associated with the formation of heterosis. In model plants *Arabidopsis* and rice, a series of candidate genes regulating heterotic traits were identified by comparative transcriptome data [12–15]. The possible relationship and interactions among these genes were uncovered, thus providing comprehensive insights into the molecular basis of heterosis. In addition to genetic regulations, more and more evidence indicated that epigenetic modifications such as DNA methylation, small RNA-mediated gene regulation and histone modification also play crucial roles in the formation of heterosis [1, 16–19]. All of these investigations have broadened the knowledge on the heterosis in plants. However, most of these studies were focused on *Arabidopsis* and various crops, such as rice, maize and sorghum. As such, the molecular basis of heterosis in other plant species remains largely unknown.

Broccoli (*Brassica oleracea* L var. *italic*) is one of the major vegetables among the *B. oleracea* varieties, and it is planted worldwide. Unlike most *B. oleracea* varieties, broccoli forms a specialized organ named curd during floral development, which composed of many flower buds and shortened inflorescence branches. The curds are the main edible organs, and the yield of curds directly determines the economic value of broccoli. Heterosis is popular in broccoli. Compared with their parents, hybrid broccoli exhibits significantly increased biomass. Especially, the curd weight of hybrids is considerably heavier than those of the parents. However, the basis of heterosis in broccoli is scarce. In the present study, transcriptome analysis was conducted. A series of genes were confirmed to show significantly differential expression levels in the hybrid broccoli and their parents, and the functions and possible relationships of the DEGs were explored. In addition, the DNA methylation patterns of the hybrids and their parents were determined. Sites with differential DNA methylation levels were also identified. These findings provided comprehensive insights into the formation of yield heterosis, and gave novel clues that could potentially assist hybrid breeding in broccoli.

## Results

### Remarkable biomass heterosis in the hybrid broccoli

**Fig. 1:**
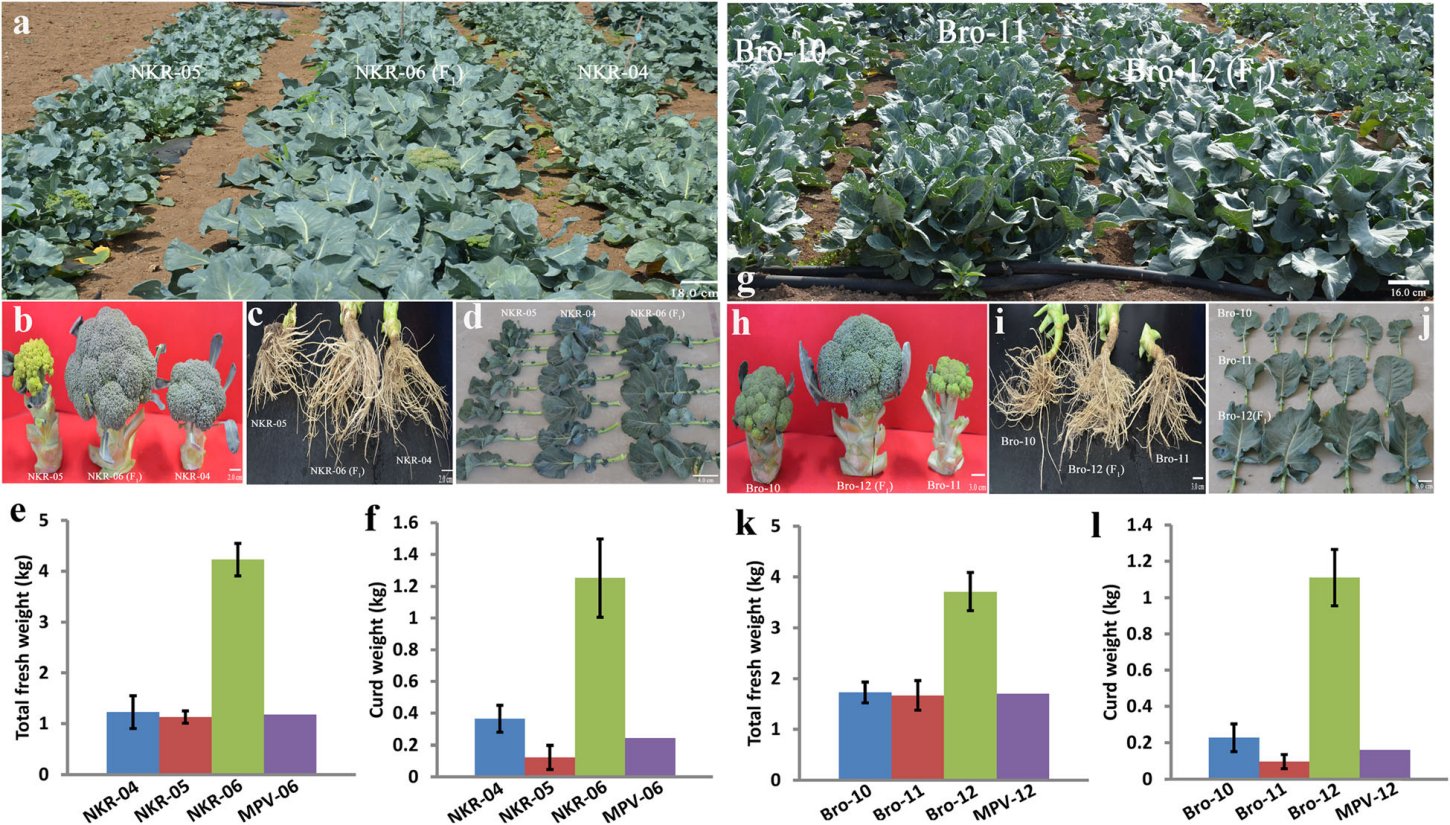
Phenotypes of the F_1_ hybrids and their parents in broccoli. **a**, **b**, **c** and (**d**) indicated the aerial organs, curds, roots and leaves of the hybrid and their parents in the NKR-04/− 05/− 06 hybrid triad, respectively. **e** and (**f**) indicated the total fresh weight and curd weight of the NKR-04/− 05/− 06 hybrid triad, respectively. **g**, **h**, **i** and (**j**) indicated the aerial organs, curds, roots and leaves of the hybrid and their parents in the Bro-10/− 11/− 12 hybrid triad, respectively. **k** and (**l**) indicated the total fresh weight and curd weight of the Bro-10/− 11/− 12 hybrid triad, respectively. MPV-06 and MPV-12 indicated the mid-parent value in the NKR-04/− 05/− 06 hybrid triad and the Bro-10/− 11/− 12 hybrid triad, respectively

Two hybrid combinations in which their F_1_ hybrids both showed obvious yield heterosis were analyzed in broccoli. Compared with their parental lines, the broccoli F_1_ hybrids displayed significantly larger curds, bigger leaves and stronger roots (Fig. 1). The total individual fresh weight in the hybrid NKR-06 was 3.6 times of the mid-parent value (MPV), and the curds of NKR-06 were 400% heavier than the MPV. Similarly, the total individual fresh weight in the hybrid Bro-12 was 120% heavier than the MPV. The weight of the curds in Bro-12 was 6.8 times of the MPV (Fig. 1). The results identified that the hybrids NKR-06 and Bro-12 demonstrated significant biomass heterosis. Specifically, the curds of both F_1_ hybrids exhibited obviously stronger heterosis in yield trait than those of their parental lines.

### More genes were actively expressed in the hybrids than those in their parents

**Fig. 2:**
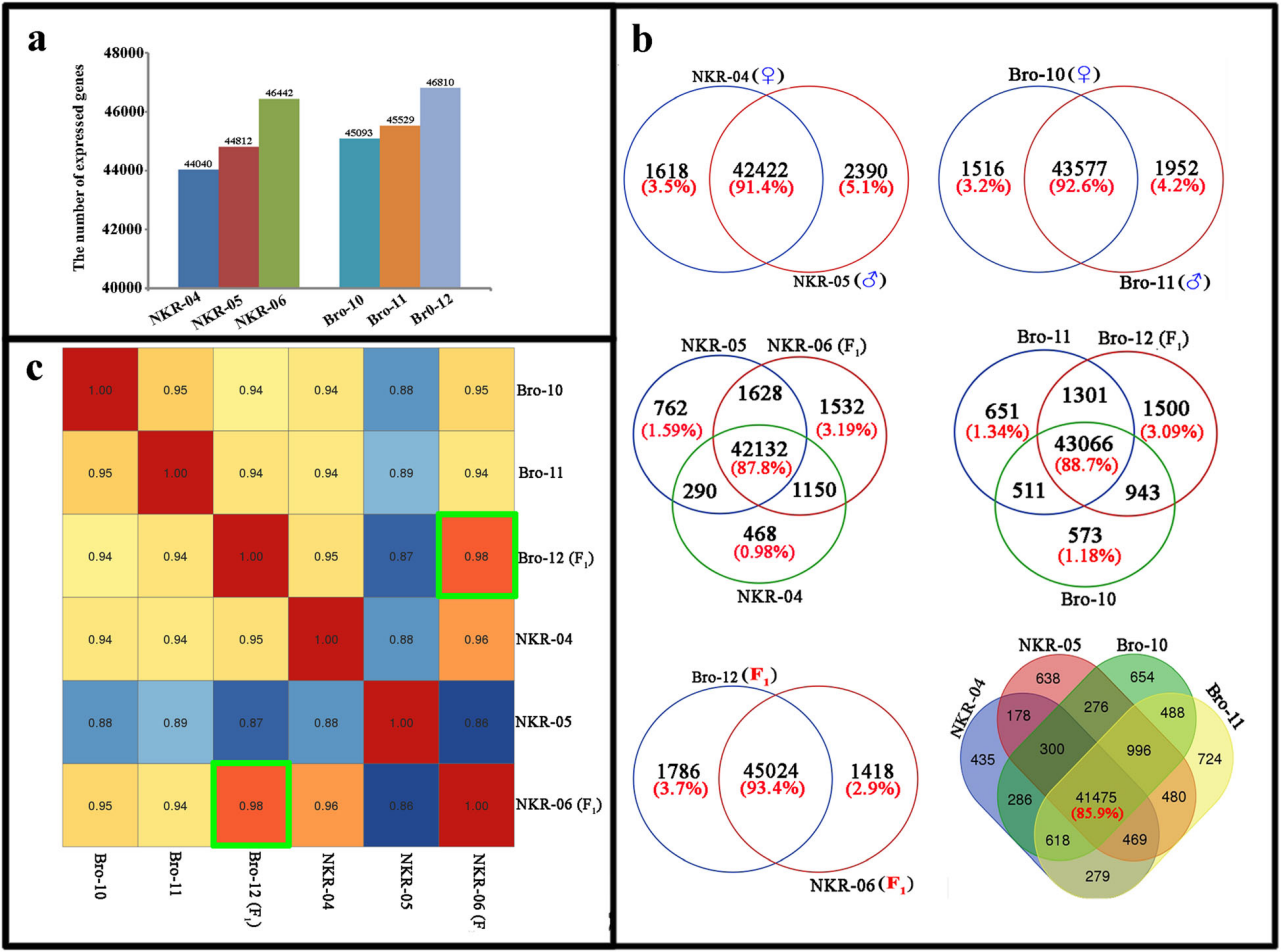
Comparative analysis of the expressed genes detected in two broccoli hybrid triads. **a** indicated the count of the expressed genes in the hybrid and their parents. **b** indicated Venn diagrams of the expressed genes drew by comparative analysis of expressed genes. **c** indicated the correlation of two hybrids and their parents calculated by the expression profiles of all detected genes in each sample. The correlation coefficient of two F_1_ hybrids was marked by green box

Over 50 million high-quality clean reads were detected in each sample, most of which were mapped to the *Brassica oleracea var. oleracea* reference genome and transcriptome (Additional file 1: Table S1). In the NKR-04/− 05/− 06 hybrid triad, 47, 962 genes showed active expression, among which 44, 040 and 44, 812 genes were detected in the parental lines NKR-04 and NKR-05, respectively. More genes (46, 442) were detected in the hybrid NKR-06 than those in the parental lines. Similarly, in the Bro-10/− 11/− 12 hybrid triad, the number of genes showing transcriptional expression in the hybrid Bro-12 (46, 810) was greater than those in the maternal line Bro-10 (45, 093) and paternal line Bro-11(45, 529). However, in each hybrid combinations, the number of actively expressed genes was similar between two parental lines (Fig. 2a, Additional file 1: Table S2). Pairwise comparison analysis further confirmed that the majority of genes (over 91%) showed common expression between the two parental lines in each hybrid triad, whereas genes showing specific expression in the paternal line were slightly greater than those in the material line (Fig. 2b). Compared with the parental lines, more genes showed specific expression in the hybrids. For example, 3.19% and 3.09% of the expressed genes specifically expressed in the hybrid NKR-06 and Bro-12, respectively. Only 0.98–1.59% of the expressed genes showed specific expression in the parental lines (Fig. 2b). In addition, although the two hybrids NKR-06 and Bro-12 generated from different hybrid combinations, more genes (93.4%) shared common expression in them than those in their parental lines (85.9%) (Fig. 2b). Moreover, correlation and clustering analysis of all of the expressed genes confirmed that the two hybrids achieved the highest correlation coefficient (*R* = 0.98) (Fig. 2c). These results indicated that although the parental lines are different, the hybridization in each broccoli hybrid combination activated more gene expression in the F_1_ hybrids compared with their parents.

### DEGs with increasing trend of expression levels were predominant

**Fig. 3:**
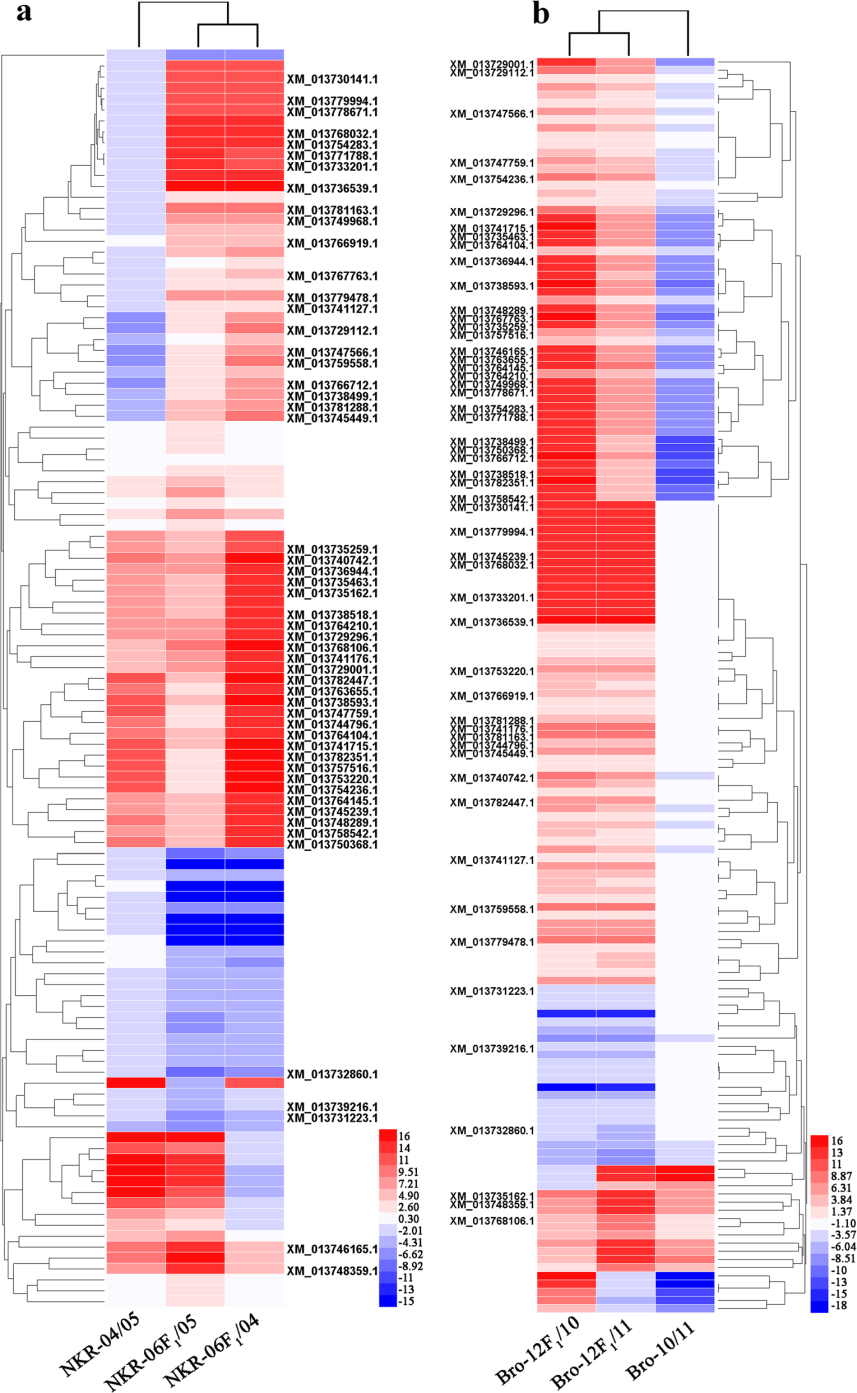
Hierarchical cluster analysis of representative DEGs in two broccoli hybrid combinations. **a** indicated the expression profiles of representative DEGs in the NKR-04/− 05/− 06 hybrid triad. **b** indicated the expression profiles of representative DEGs in the Bro-10/− 11/− 12 hybrid triad. The expression levels of these DEGs in the hybrid were significantly different from those in both parents. The accession numbers of DEGs which shared in both hybrid triads were visual. The heat maps were constructed based on the log_2_ (fold-change of the normalized expression levels) of two arbitrary samples in each hybrid triad. The color scale represents the log_2_ (fold-change of the normalized expression levels) of two arbitrary samples with blue denoting low expression and red denoting high expression

Besides the count of actively expressed genes in two hybrid triads, the differential expression profiles of genes were further studied. In the NKR-04 and NKR-05 hybrid combination, 789 genes displayed significantly differential expression levels between the two parental lines (corrected *p*-value < 0.05). Among them, the proportion of genes showing significantly higher and lower expression levels in the maternal line NKR-04 than those in the paternal line NKR-05 was similar (Table 1). However, the DEGs between the hybrid NKR-06 and either of the parental lines were obviously less than those detected in two parents (Table 1). In total, 936 genes showed significantly differential expression levels between the hybrid NKR-06 and the parents (corrected p-value < 0.05), among which most of the DEGs (over 80%) showed higher expression levels in the hybrid NKR-06 than those in its parents. Moreover, the expression levels 115 of the 936 genes were significantly different from those in both parents. Compared with the parents, the majority of the 115 genes (79 out of 115) showed higher expression levels in the hybrid NKR-06 (Table 1, Fig. 3a and Additional file 2: Data set 1). Similarly, the DEGs between the two parental lines Bro-10 and Bro-11 were considerably greater than those detected between the hybrid Bro-12 and either of its parental lines. In total, 932 DEGs were detected between the hybrid Bro-12 and its parents, of which the majority of the DEGs showed higher expression levels in the hybrid than those in the parents. 153 of the 932 DEGs showed significantly higher expression levels in the hybrid Bro-12 than those in its parents, and most of them increased expression levels in the hybrid (Table 1, Fig. 3b, Additional file 2: Data set 2). Among the 115 DEGs detected in the NKR-04/− 05/− 06 hybrid triad and the 153 DEGs detected in the Bro-10/− 11/− 12 hybrid triad, 53 DEGs were shared in both hybrid triads. The majority of the 53 DEGs (50 out of 53) showed significantly higher expression levels in the hybrids than those in their parents (Fig. 3, Additional file 2: Data set 3). These results further confirmed that the hybridization not only activated more gene expression in the broccoli F_1_ hybrids, but also made more genes displayed significantly higher expression levels in the hybrids than those in their parents.

**Table 1:** Differentially expressed genes detected in the hybrid broccoli and their parents

Hybrid set	DG_PP_ or DG_HP_				
	Samples	Up	Down	B2P	Total
NKR-04 x NKR-05	P04 vs. P05	425 (53.3%)	373 (46.7%)		798
	F06 vs. P04	460 (80.6%)	111(19.4%)		571
	F06 vs. P05	388 (80.8%)	92 (19.1%)		480
	F06 vs. (P04 and P05)	79 (68.7%)	26 (22.6%)	10 (8.7%)	115
Bro-10 x Bro-11	P10 vs. P11	472 (52.7%)	424 (47.3%)		896
	F12 vs. P10	418 (73.6%)	150 (26.4%)		568
	F12 vs. P11	416 (80.5%)	101(19.5%)		517
	F12 vs. (P10 and P11)	123 (80.4%)	22 (14.4%)	8 (5.2%)	153

Notice: *DG*_*PP*_ indicated the differentially expressed genes, (*DEGs*) between two parents, *DG*_*HP*_ indicated the *DEGs* between the hybrid and their parents, *B2P* indicated the expression levels of the *DEGs* between the two parental lines. P04, P05, F06, P10, P11 and P12 indicated NKR-04, NKR-05, NKR-06, Bro-10, Bro-11 and Bro-12, respectively

### Few genes showed non-additive expression patterns

**Fig. 4:**
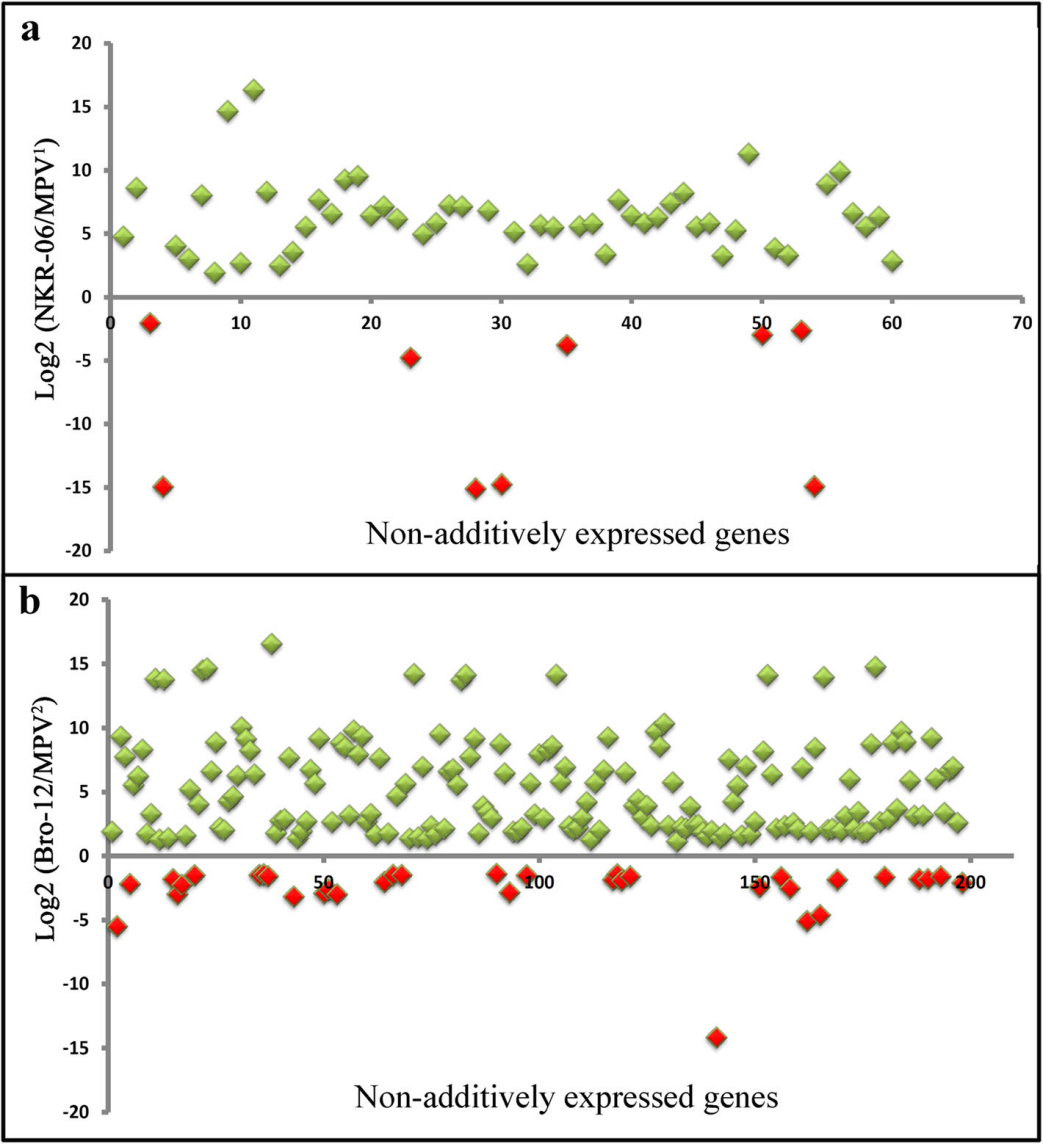
Expression patterns of non-additively expressed genes directed in two hybrid combinations. **a** indicated the expression patterns of non-additively expressed genes in the NKR-04/− 05/− 06 hybrid triad. **b** indicated the expression patterns of non-additively expressed genes in the Bro-10/− 11/− 12 hybrid triad. The *X* axis indicated that serial number of non-additively expressed genes marked in Supplementary Data set 4. The *Y* axis indicated the relative expression levels of the hybrid compared with the MPV. MPV^1^ indicated the mid-parent expression level of genes in the NKR-04/− 05/− 06 hybrid triad; MPV^2^ indicated the mid-parent expression level of genes in the Bro-10/− 11/− 12 hybrid triad. Green boxes indicated that the expression levels of genes were higher than the MPV. Red boxes indicated that the expression levels of genes were lower than the MPV

Genes in the hybrid having the expression levels significantly deviated from the MPV were designated as non-additively expressed genes. In the NKR-04/− 05/− 06 hybrid triad, among the 47, 962 genes only 60 genes (0.13%) showed non-additive expression patterns (Additional file 2: Data set 4), implying that the vast majority of the genes in this hybrid triad exhibited additive expression patterns. Further analysis indicated that 51 out of the 60 non-additively expressed genes (85%) exhibited higher expression levels than the MPV (Fig. 4a, Additional file 2: Data set 4). Similarly, a small proportion of genes (198/48, 545 = 0.4%) in the Bro-10/− 11/− 12 hybrid triad showed non-additive expression patterns (Additional file 2: Data set 4). Among the 198 non-additively expressed genes, the expression levels of 163 genes (83%) were higher than the MPV (Fig. 4b). In total, 258 genes displayed non-additive expression patterns, among which 37 genes were shared in the two hybrid triads. Moreover, the expression levels of 35 out of the 37 genes were significantly higher than those of their two parents (Additional file 2: Data set 4). These results demonstrated that the majority of genes exhibited additive expression patterns in the two hybrid triads. In the non-additively expressed genes, genes exhibiting higher expression levels than the MPV were predominant.

### Gene regulatory networks involved in light, hormone and stress responses were altered in the hybrids

**Fig. 5:**
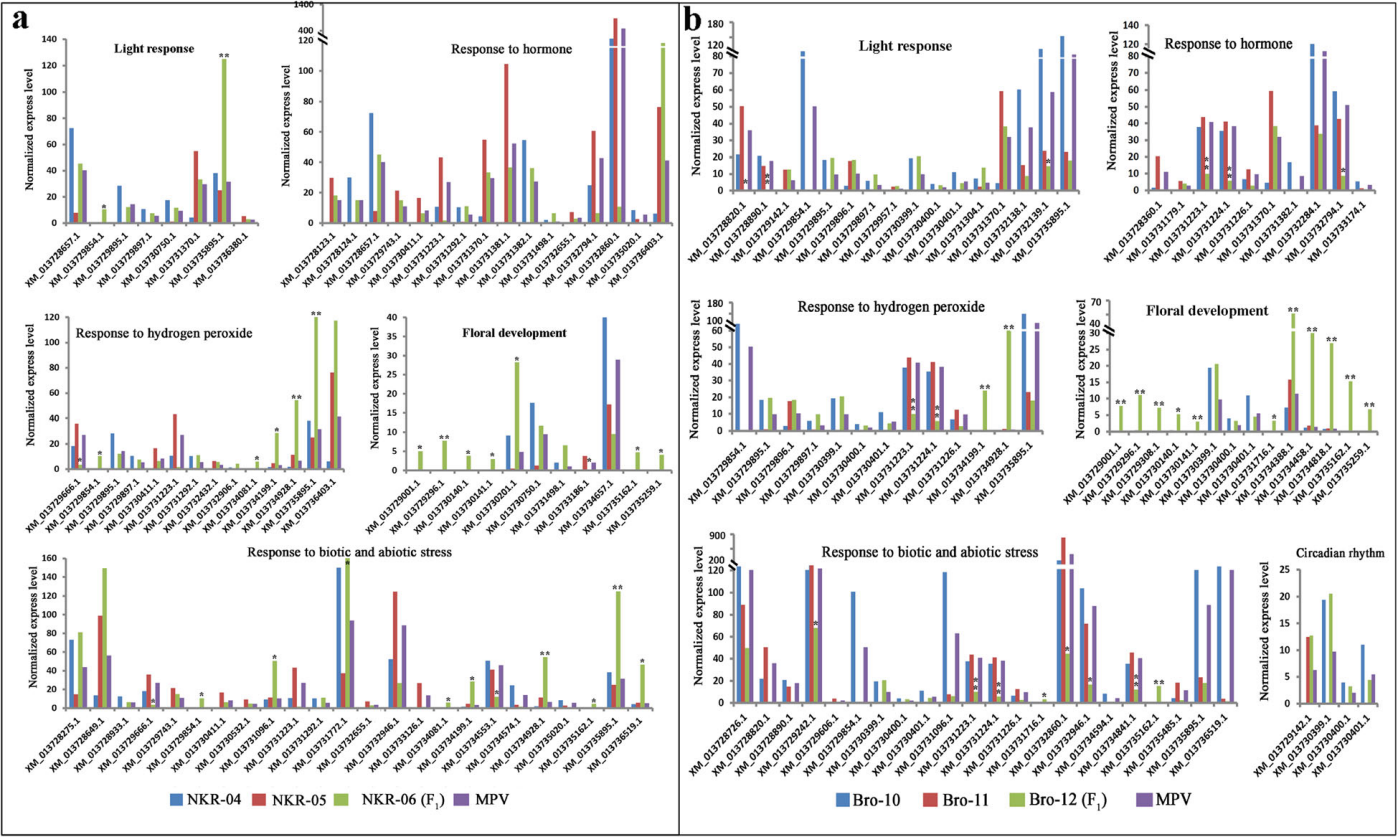
Expression levels of genes involved in several overrepresented biological processes. **a** and (**b**) indicated the expression levels of genes involved in light, hormone, hydrogen peroxide and stress responses, floral development and circadian rhythm in the NKR-04/− 05/− 06 hybrid triad and the Bro-10/− 11/− 12 hybrid triad, respectively. * indicated the relative expression levels of the genes in the hybrid were different from the MPV (*p*-value < 0.05). ** indicated the relative expression levels of the genes in the hybrid were significantly different from the MPV (corrected p-value < 0.05). The functional annotations of these genes were showed in Supplementary Table S2

All DEGs were functional classification by gene ontology (GO) analysis (Additional file 2: Data set 5 and Data set 6). GO terms showed significantly enriched in biological processes were predominant, which were further clustered by ReviGO. The results indicated that GO terms involved in responses to environmental stimuli in vivo and vitro, such as “light response”, “response to hormone”, “response to biotic and abiotic stress”, “response to hydrogen peroxide” and “water and ion homeostasis”, were significantly overrepresented. In light response processes, the majority of GO terms were related to red light signaling pathways, followed by blue light signaling pathways, and response to light intensity and UV. Abscisic acid, jasmonic acid, salicylic acid, brassinosteroid and auxin-mediated signaling pathways were predominant in hormone response processes. Signaling pathways involved in response to various biotic and abiotic stresses were overrepresented in stress response processes. Responses to water deprivation and ion transport, especially iron, cadmium and manganese transport, were overrepresented in GO terms involved in water and ion homeostasis (Table 2, Additional file 2: Data set 5 and Data set 6). Gene expression profile assay indicated that the majority of the DEGs targeting these overrepresented response processes exhibited higher expression levels in the hybrids than those in both or either of the parents, except for the DGEs identified in the “response to biotic and abiotic stress” process (Fig. 5).

**Table 2:** Overrepresented biological processes involved in the DEGs detected in two broccoli hybrid triads

Overrepresented biological processes	No. of enriched GO terms	
	NKR-04/−05/−06 triad	Bro-10/−11/−12 triad
light response	14	24
response to hormone	14	15
response to biotic and abiotic stress	23	19
response to hydrogen peroxide	10	6
water and ion homeostasis	15	12
floral development	8	8
shoot and leaf development	5	4
root development	4	5
embryo development	2	4
cell division and differentiation	4	6
cell wall biosynthesis	3	2
Photosynthesis/chloroplast development	6	5
fatty acid biosynthesis and metabolism	8	7
sugar biosynthesis and metabolism	7	7
carbohydrate metabolism	2	4
protein syhthesis and metabolism	16	19
DNA/RNA syhthesis and metabolism	34	31
anthocyanin and flavonoid biosynthesis	3	5
regulation of circadian rhythm	0	4
others	19	33

### Gene regulation networks involved in organ growth and development were also altered in the hybrids

In addition to gene regulation networks involved in responses to environmental stimuli, which were significantly adjusted in the hybrids, a large proportion of the GO terms targeted by these DEGs displayed prominently enriched in pathways associated with growth and development, such as “leaf, root and shoot growth and development”, “floral development”, “embryo development”, “cell division and differentiation” and“photosynthesis and chloroplast development” (Table 2, Additional file 2: Data set 5 and Data set 6). Among them, GO terms involved in floral development were mainly targeted by the DEGs, which exhibited significantly differential expression levels in the hybrid compared with both parents, and/or non-additive expression patterns. The majority of these genes were annotated to cytochrome P450, type III polyketide synthases, glucan endo-1, 3-beta-glucosidase, fatty acyl-CoA reductase, non-specific lipid-transfer proteins, lipid-transfer proteins, AT-hook motif nuclear-localized proteins, Tetraketide alpha-pyrone reductase, 4-coumarate-CoA ligase, alpha-dioxygenase, monocopper oxidase, ubiquitin-related proteins, tetraketide alpha-pyrone reductase and tapetum-specific protein, which played important roles in floral development (Additional file 2: Data set 3). Another prominently overrepresented biological processes involved in growth and development were “biosynthesis and metabolism of sugar, fatty acid and carbohydrate” and “DNA/RNA and protein synthesis and modification” (Table 2, Additional file 2: Data set 5 and Data set 6), implying that the biosynthesis and metabolism processes in the hybrid broccoli were more active than those in their parents. Gene expression profile assay also indicated that the majority of the DEGs targeting these overrepresented growth and development processes exhibited higher expression levels in the hybrids than those in both or either of the parents (Additional file 3: Figure S1).

### Expression patterns of DEGs were confirmed by qRT-PCR

**Fig. 6:**
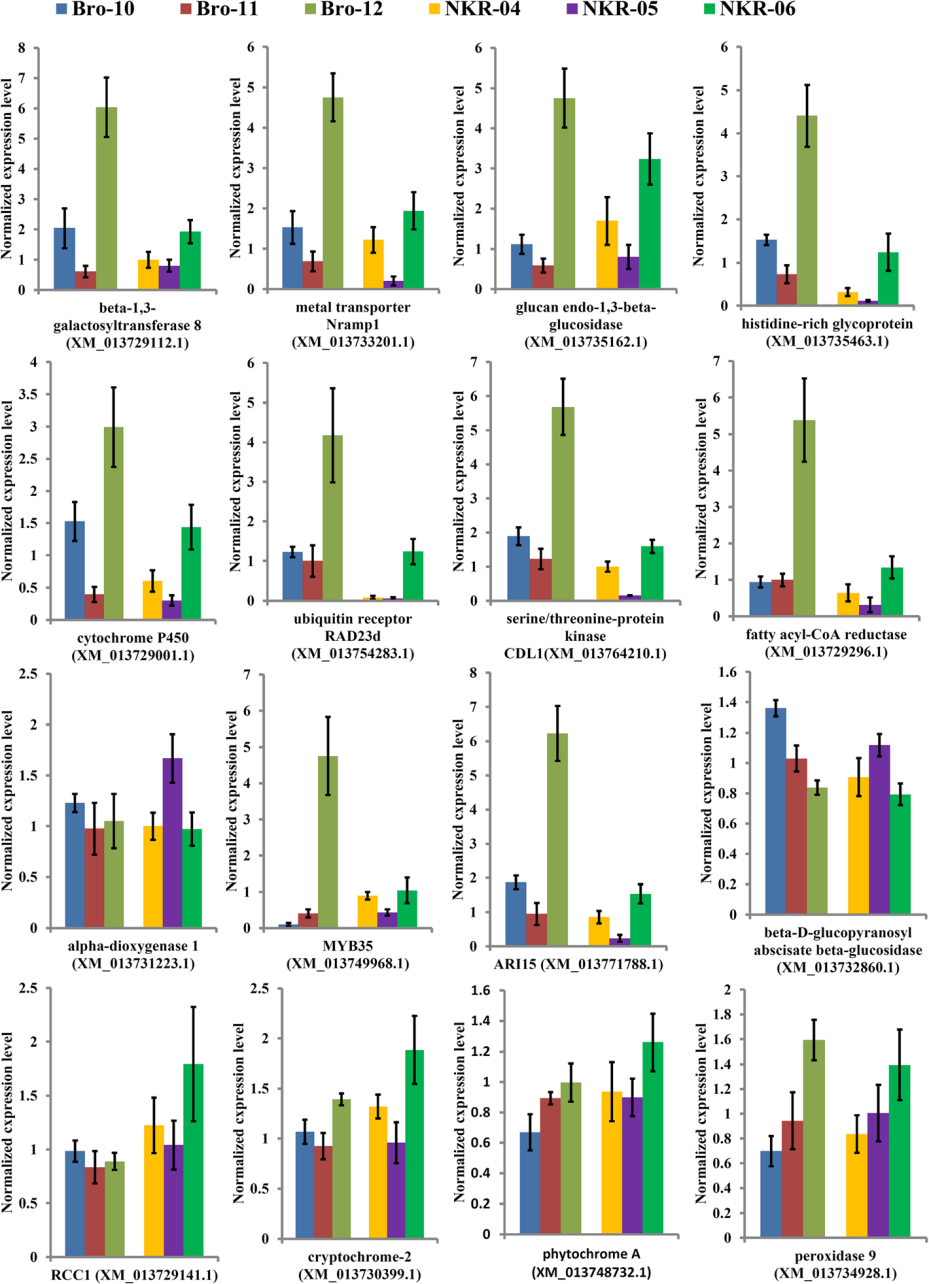
Quantitative expression analysis of representative differentially expressed genes detected in two hybrid triads by qRT-PCR

Sixteen genes that showed significantly differential expression levels in the hybrids and their parents were selected to conduct quantitative real-time RT-PCR (qRT-PCR) identification. The expression patterns of 15 out of the 16 DEGs detected by qRT-PCR were highly consistent with the data produced by high-throughput sequencing (Fig. 6). In addition, the expression patterns of eight DEGs, including *fatty acyl-CoA reductase*, *peroxidase 9*, *phytochrome A*, *cytochrome P450*, *glucan endo-1,3-beta-glucosidase A6*, *metal transporter Nramp1*, *alpha-dioxygenase 1*, and *beta-D-glucopyranosyl abscisate beta-glucosidase*, were further confirmed in another hybrid triad of broccoli (i.e., BNR-M (male), BNR-F (female) and BNR-H (F_1_ hybrid)). The F_1_ hybrid BNR-H also exhibited remarkable curd yield heterosis. All eight genes displayed similar expression patterns in the BNR-M/-F/-H hybrid triad to those in the NKR-04/− 05/− 06 and the Bro-10/− 11/− 12 hybrid triads (Additional file 3: Figure S2).

### F_1_ hybrids showed higher DNA methylation ratio than their parents

**Fig. 7:**
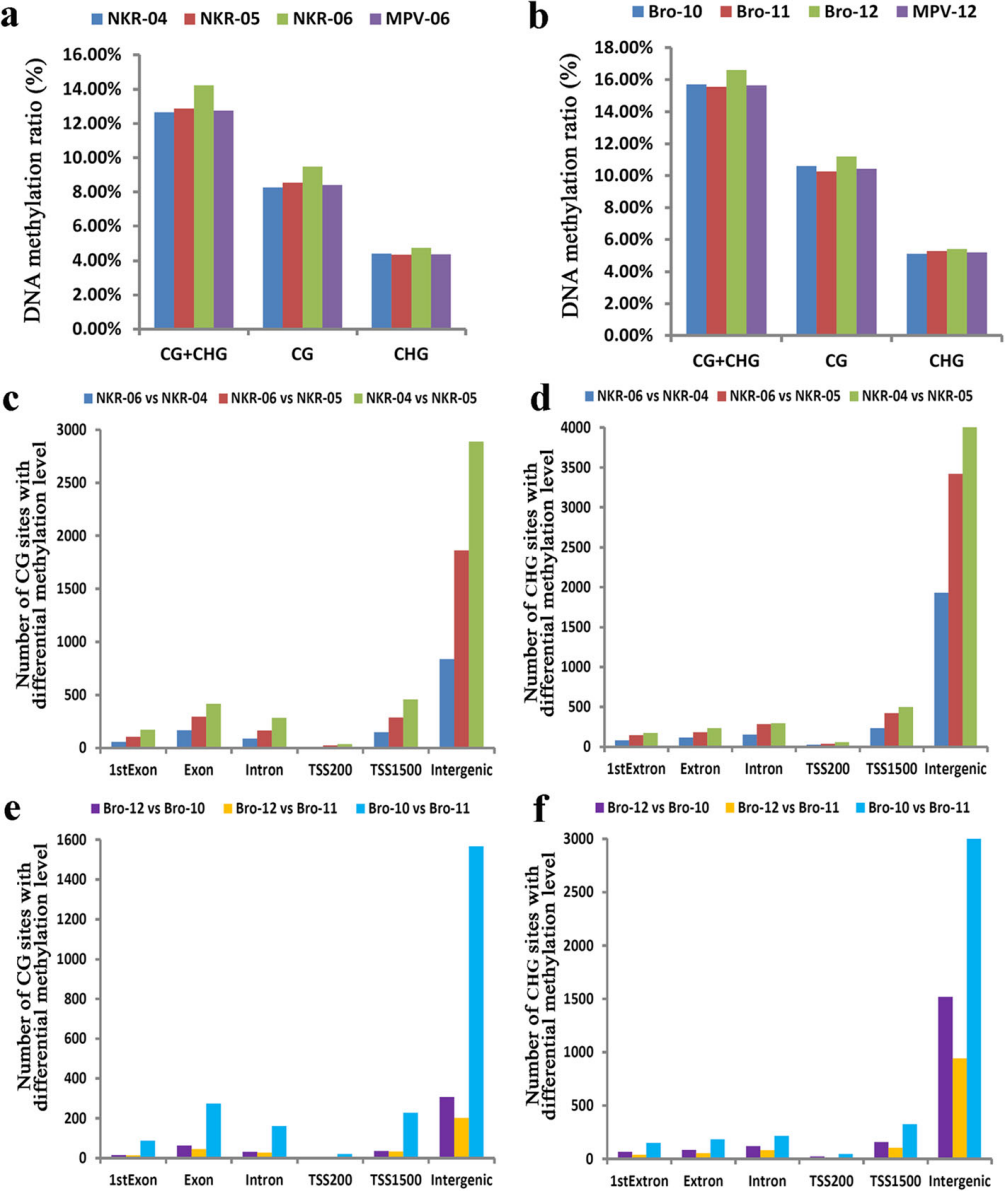
DNA methylation ratio and distributions of CG and CHG sites with differential methylation levels. **a** and (**b**) indicated the genomic DNA methylation ratio at CG and CHG sites in the NKR-04/− 05/− 06 hybrid triad and the Bro-10/− 11/− 12 hybrid triad, respectively. **c** and d indicated the distributions of CG (**c**) and CHG (**d**) sites with differential methylation levels in the NKR-04/− 05/− 06 hybrid triad; **e** and f indicated the distributions of CG (e) and CHG (**f**) sites with differential methylation levels in the Bro-10/− 11/− 12 hybrid triad. 1^st^Exon, Exon, intron, TSS200 and TSS1500 indicated the regions of the first exon, the whole exons of genes, the whole introns of genes, the upstream 200 bp of the transcription termination site (TSS) and the upstream 1500 bp of TSS, respectively. “intergenic” indicated the intergenic regions

**Fig. 8:**
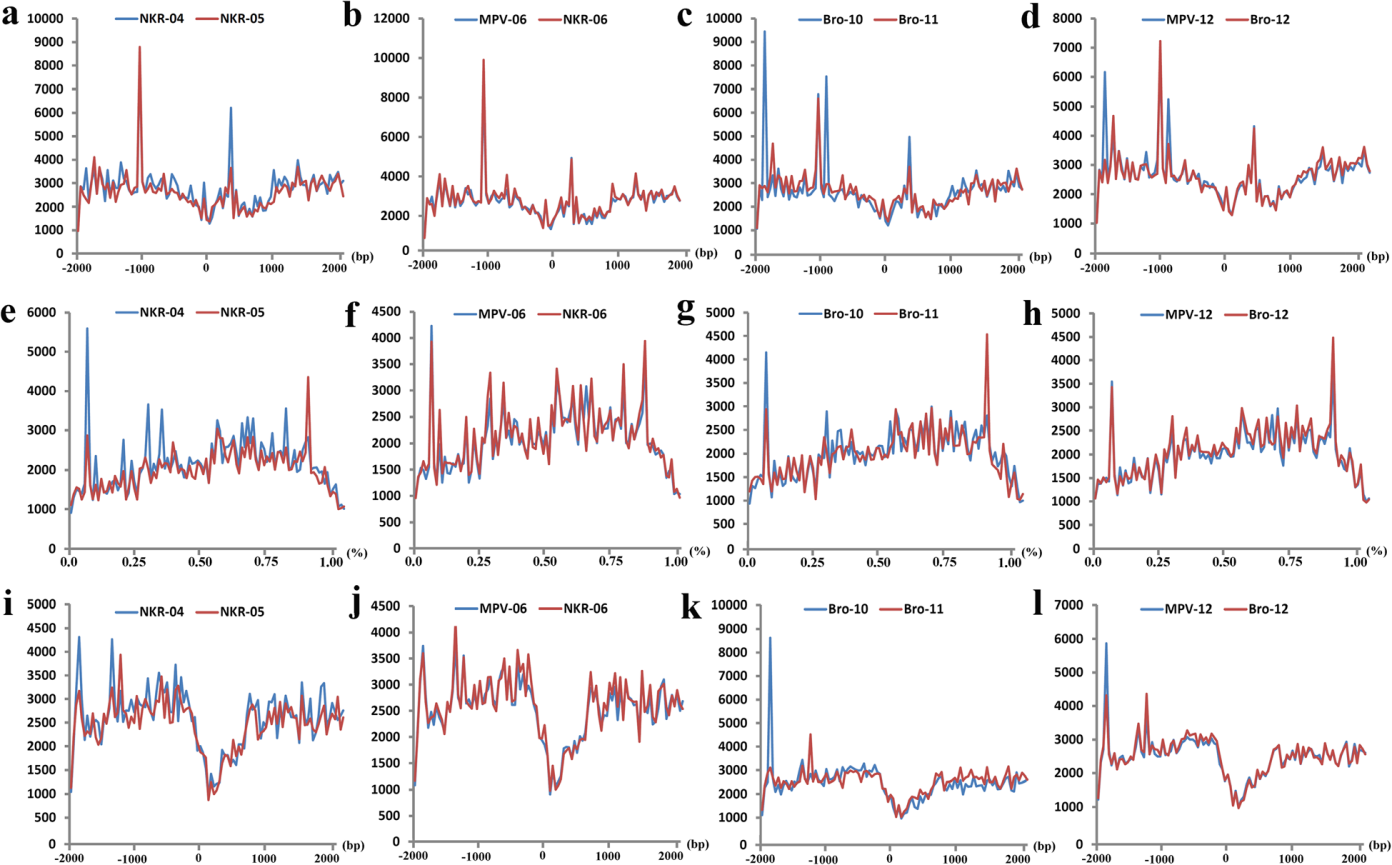
DNA methylation levels at the CG sites in different regions of genes. **a**, **b**, **c** and (**d**) indicated the relative DNA methylation levels at CG sites of transcription start site (TSS) regions. **e**, **f**, **g** and (**h**) indicated the relative DNA methylation levels at the CG sites of gene coding regions. **i**, **j**, **k** and (**l**) indicated the relative DNA methylation levels at the CG sites of transcription termination site (TTS) regions. *X* axis showed the ±2000 bp of TSS **a**, **b**, **c** and (**d**), the relative position of gene body **e**, **f**, **g** and (**h**) and the ±2000 bp of TTS **i**, **j**, **k** and (**l**), respectively. *Y* axis showed the relative DNA methylation level. MPV-06 and MPV-12 indicated the mid-parent value of relative DNA methylation level in the NKR-04/− 05/− 06 hybrid triad and the Bro-10/− 11/− 12 hybrid triad, respectively

The DNA methylation at the two popular sites (i.e., CG and CHG. H = T or A) in the whole genomes of the two hybrids and their parents were analyzed by using MethylRAD method (Additional file 2: Data set 7). The total DNA methylation ratios (methylated CG and CHG sites/total CG and CHG sites) were 14.23%, 12.66% and 12.87% in NKR-06, NKR-04, and NKR-05, respectively. The total DNA methylation ratios were 16.60%, 15.70%, and 15.54% in Bro-12, Bro-10, and Bro-11, respectively (Fig. 7a, b, Additional file 1: Table S3). These data confirmed that the hybrids had slightly higher total DNA methylation ratio than their corresponding parents as well as the MPV (Fig. 7a, b). In addition, the data indicated that the DNA methylation ratio at the CG sites was higher than that at the CHG sites in each chromosome, except for the chromosome 9 of the hybrid Bro-12 (Additional file 1: Table S3). However, the distribution patterns of most methylated sites at the different elements of genomes were similar in the hybrids and their parents. Both the CG and CHG sites at the intergenic regions appeared to easily occur methylation, followed by the regions at the gene body, TSS1500 (upstream 1500 bp of transcription start sites (TSS)), intron, 1^st^exon and TSS200 (upstream 200 bp of TSS) (Additional file 3: Figure S3). Among them, the DNA methylation levels of the gene regions, including the TSS, the gene coding region, and the transcription termination site (TTS) were explored. The results confirmed that although the DNA methylation levels differed between the two parents of each hybrid triad, the DNA methylation levels of the hybrids at either the CG sites or the CHG sites in the gene regions were similar to the MPV (Fig. 8, Additional file 1: Table S4, Additional file 3: Figure S4).

### Sites exhibiting differential methylation levels between the parents were greater than those between the hybrids and the parents

In the NKR-04/− 05/− 06 hybrid triad, compared with the parental lines NKR-04 and NKR-05, 1, 304 and 2, 732 CG sites showed significantly differential methylation levels in the hybrid NKR-06, respectively. These sites represented 464 and 783 genes with significantly methylation levels, respectively. By contrast, 4, 254 CG sites exhibited significantly differential methylation levels between two parents, which made 1, 322 genes show significantly differential methylation levels. At the CHG sequence regions, 2, 536 and 4, 492 sites exhibited significantly differential methylation levels between NKR-06 and NKR-04, and between NKR-06 and NKR-05, indicating that 296 and 514 genes showed significantly differential methylation levels, respectively. However, as many as 5, 355 CHG sites exhibited significantly differential methylation levels between the parents NKR-04 and NKR-05. These sites represented 664 genes showing significantly differential methylation levels (Additional file 2: Data set 8 and Data set 9). Similarly, in the Bro-10/− 11/− 12 hybrid triad, the sites and genes showing significantly differential methylation levels between two parents were greater than those between the hybrid and each of the parental line (Additional file 2: Data set 10 and Data set 11).

### Sites with differential methylation levels were predominant in the intergenic regions

The distributions of the sites with differential methylation levels were explored. The data indicated that between the parents or between the hybrid and the parents, the sites with differential methylation levels were mostly enriched in the intergenic regions, followed by the gene coding regions (1^st^Exon + other extrons), the TSS1500 (upstream 1500 bp of TSS), intron and TSS200 (upstream 200 bp of TSS) (Figs. 7c-f, Additional file 1: Table S5).

### Few DEGs exhibited differential methylation levels

**Fig. 9:**
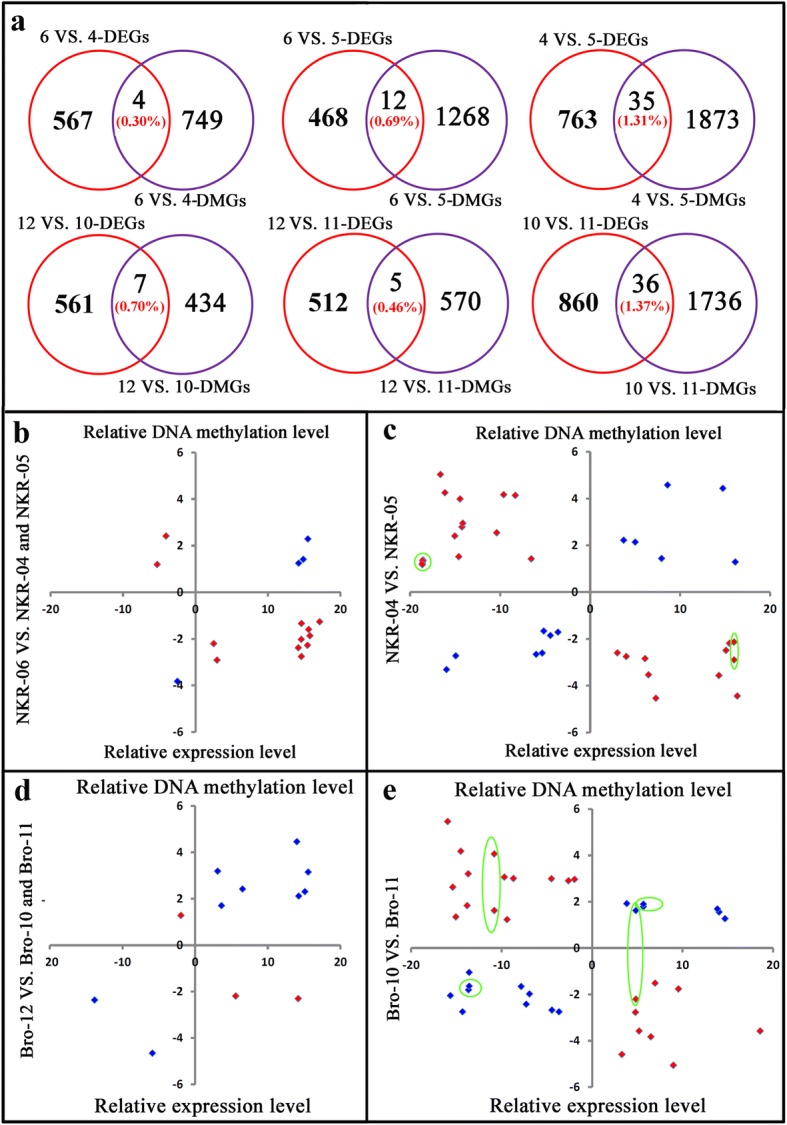
Relationship of differential expression levels of genes and their DNA methylation levels. **a** indicated the Venn diagrams of genes showing differential expression levels and/or differential DNA methylation levels by pairwise comparison analysis. DEGs indicated the differentially expressed genes. DMGs indicated the differentially methylated genes. 4, 5, 6, 10, 11 and 12 indicated NKR-04, NKR-05, NKR-06, Bro-10, Bro-11 and Bro-12, respectively. **b**, **c**, **d** and (**e**) indicated the relationship of the expression levels of DEGs and their DNA methylation levels. The red colors indicted the expression levels of DEGs were negatively correlated with their DNA methylation levels at the CG and/or CHG sites. The blue colors indicated that the expression levels of DEGs were positively correlated with their DNA methylation levels at the CG and/or CHG sites. *X* axis indicated the relative expression levels of genes. *Y* axis indicated the relative DNA methylation levels of genes. The DEGs which showed differentially DNA methylation levels at the CG sites as well as the CHG sites were marked by green circles

The relationships between the differential expression levels of genes and their DNA methylation levels were analyzed. In the NKR-04/− 05/− 06 hybrid triad, 753 genes showed significantly differential methylation levels between NKR-06 and NKR-04. Genes with significantly differential methylation levels between NKR-06 and NKR-05 were 1, 280. Between two parents, 1, 908 genes showed significantly differential methylation levels. Comparative transcriptome analysis indicated that 571 genes showed differential expression levels between NKR-06 and NKR-04. 480 and 798 genes showed differential expression levels between NKR-06 and NKR-05, and between two parents, respectively. However, compared with NRK-04 and NKR-05, only four and 12 genes simultaneously showed differential expression levels and DNA methylation levels in the hybrid NKR-06, respectively. Between NRK-04 and NKR-05, 35 DEGs showed differential methylation levels. Similarly, in the Bro-10/− 11/− 12 hybrid triad, only seven, 5 and 36 genes simultaneously exhibited differential methylation levels and expression levels between Bro-12 and Bro-10, between Bro-12 and Bro-11, and between two parents Bro-10 and Bro-11, respectively (Fig. 9, Additional file 1: Table S6). Among these DEGs which also exhibited differential methylation levels, a large proportion of them (60 DEGs) appeared to be negatively correlated with their DNA methylation levels. However, there are over 40 DEGs that their expression levels trended to show positively correlated with their DNA methylation levels (Fig. 9, Additional file 1: Table S6).

## Discussion

Transcriptome and other omics have provided powerful strategies for elucidating the molecular basis of heterosis in diverse plants [12, 15, 20]. In the present study, the transcriptome data confirmed that the hybridization activated more genes in the hybrid broccoli with obviously yield heterotic trait than those in their parents. However, the whole gene expression profiles of the hybrids and their parents were similar. Only a small proportion of the genes exhibited significantly differential expression levels in the hybrids and their parents. Investigations involved in the heterosis produced by intraspecific hybridization in *Arabidopsis*, rice and other plant species also confirmed that the majority of genes showed similar expression profiles in the hybrids and their parents, and that the expression levels of most genes were near the MPV [12, 21, 22]. These findings implied that extensive and dramatic changes of the whole gene expression profiles may not be required in the formation of certain heterotic traits through intraspecific hybridization, although the hybridization could make some genes activate or inhibit expression in the hybrids. The additive expression balance of most genes in the hybrids establishes an important foundation for the formation of heterosis. Nevertheless, the genes exhibiting significantly differential expression profiles in the hybrids and their parents, especially the non-additively expressed genes, provided valuable clues to elucidate the formation of heterotic traits. The functional annotation indicated that a large proportion of the DEGs were involved in response processes of environmental stimuli. Among them, “light response”, “response to hormone”, “response to biotic and abiotic stress” and “response to hydrogen peroxide” were overrepresented. The hormone-mediated signaling pathways targeted by the DEGs were mainly involved in abscisic acid, jasmonic acid, salicylic acid, brassinosteroid and auxin. All these hormones have been demonstrated to play important roles in the formation of heterosis [23–27]. Pathways involved in multiple biotic and abiotic stresses were also confirmed to involve in the heterosis [25, 28, 29]. These findings indicated that the DEGs involved in hormone-mediated regulation pathways and biotic and abiotic stresses should also play important roles in the formation of heterosis of broccoli. Interestingly, some DEGs were confirmed to function in light response and hydrogen peroxide response, which were few reported to be associated with the heterosis in plants. Light is not only the energy of photosynthesis but also an important signal regulator to function in photomorphogenesis and other growth and development processes [30–32]. A series of GO terms, such as “response to light intensity”, “response to red or far red light”, “detection of visible light” and “response to blue light”, displayed significantly enriched in light-mediated response processes in present study. Remarkably, a recent report indicated that a key factor of light signaling networks, *PIF4*, could contribute to heterosis by regulating the auxin pathway [24]. In addition, hydrogen peroxide is an important signaling molecule. It can interplay with diverse phytohormone signals, such as abscisic acid, salicylic acid, jasmonic acid, auxin and brassinosteroid, to regulate plant developmental processes and stress responses [33–35]. Recent reports confirmed that hydrogen peroxide could induce the oxidation of the *BZR1* transcription factor, a master regulator of brassinosteroid signal pathway. Oxidative modification enhances *BZR1* transcriptional activity by promoting its interaction with key regulators in the auxin signal pathway and light signal pathway, including *ARF6* and *PIF4* [36]. In the present study, multiple pathways involved in hydrogen peroxide response were significantly adjusted in the hybrid broccoli. These findings suggested that besides processes involved in hormone responses, and stress responses, light and hydrogen peroxide-mediated signaling pathways may represent two novel classes of regulatory processes that function in yield heterosis in broccoli. Among them, hydrogen peroxide as a key signaling molecule should play crucial roles in the formation of yield heterosis of broccoli by interplaying with diverse hormone-mediated signaling pathways as well as light signaling pathway.

Besides the response processes of environmental stimuli, several pathways involved in growth and development, such as “leaf, root and shoot growth and development”, “floral development”, “embryo development” and “cell division and differentiation”, also exhibited overrepresented in the GO terms targeted by the DEGs. Among them, the overrepresented pathways involved in floral development were mainly targeted by those DEGs, which exhibited significantly differential expression levels in the hybrid broccoli compared with both parents, and/or non-additive expression patterns. In total, 53 such DEGs, including 35 non-additively expressed genes, were identified in two broccoli hybrid triads. 13 out of the 53 DEGs were confirmed to play important roles in the anther and/or pollen development. For example, *CYP703A2* and *CYP704B1* are essential for the sporopollenin formation in diverse plant species [37–39]. *Acyl-CoA Synthetase 5* (*ACOS5*) encodes an acyl-CoA synthetase protein that is required for sporopollenin monomer biosynthesis [28, 40]. Plant specific type III polyketide synthase (PSK) genes, *PSKA* and *PSKB*, encode hydroxyalkyl a-pyrone synthases to catalyze the condensation of malonyl-CoA units with various CoA ester starter molecules that are essential for the pollen development [41, 42]. *Tetraketide alpha-pyrone reductase 1* (*TKPR1*) and *TKPR2* are confirmed to function together with *ACOS5* and *PKSA/B* to hydroxylate a-pyrone compounds that are important sporopollenin precursors of pollen development [43]. *ABC transporter G family member 26* (*ABCG26*), an ATP binding cassette transporter, plays crucial roles in *Arabidopsis* pollen exine formation and male fertility [44, 45]. The *transposable element silencing* via *AT-hook* (*TEK*) encodes an AT-hook nuclear localized (AHL) protein in *Arabidopsis* that is highly homologous with the *AHL16* in broccoli. *TEK* is highly expressed in tapetum during the tetrad stage and determines nexine formation in the pollen wall [46]. Moreover, the data indicated that all of these DEGs involved in floral development showed significantly over-parental expression patterns in the hybrids. The curd of broccoli mainly composed of flower buds. Accordingly, it should speculate that significantly increased expression levels of these genes could promote the growth and development of the flower organ, and therefore may function in the formation of yield heterosis of curds in the hybrid broccoli.

Genetic diversity even very small difference between two parents may be crucial but not the only factor affecting the formation of heterotic traits in F_1_ hybrids. Epigenetic modification, such as DNA methylation, has been confirmed to function in this phenomenon [15, 47, 48]. The data indicated that the hybridization made more sites facilitate cytosine methylation in the hybrid broccoli. However, the DNA methylation levels of most sites in the hybrids were near the MPV, and showed an additive character. The sites with differential methylation levels were predominant in the intergenic regions. Only few DEGs simultaneously exhibited differential methylation levels. In *Arabidopsis*, the heterotic hybrids showed increased DNA methylation ratios compared with the parents. The increased DNA methylation of the hybrid genomes also predominantly occurred in the non-protein-coding regions. The regions with different DNA methylation patterns were typically covered by small RNAs, implying that the RNA-directed DNA methylation (RdDM) pathway directed DNA methylation in the hybrids and play an important role in heterosis [49]. The distributions of small RNAs were not explored in the present study. Nevertheless, it should speculate that the remodeling of DNA methylation directed by the non-coding RNAs should play roles for the formation of curd yield heterosis in broccoli. Further investigations are required to elucidate how these different DNA methylation sites and their relationship with gene expression function in this phenomenon.

## Conclusions

The gene expression profiles and DNA methylation patterns of the hybrid broccoli and their parents were uncovered. The gene regulatory networks involved in signaling pathways of light, hormone and hydrogen peroxide, responses to stresses, and regulation of floral development were identified to play crucial roles in the yield heterosis of broccoli. Moreover, the present investigation indicated that the light and hydrogen peroxide-mediated signaling pathways represent two novel classes of regulatory processes that could function in yield or biomass heterosis of plants. Totally, 53 candidate genes closely involved in curd yield heterosis were identified. In addition, the data confirmed that the sites with differential methylation levels were predominant in the intergenic regions of hybrid broccoli. The changes of DNA methylation levels in gene regions did not significantly affect their expression levels. The remodeling of DNA methylation mainly directed by the non-coding RNAs then was speculated to also play roles in the formation of heterotic trait of the hybrid broccoli. These findings provided comprehensive insights into the curd yield heterosis in broccoli, and were significant for breeding high-yield broccoli varieties.

## Methods

### Plant materials

Homozygous broccoli parental lines (NKR-04, NKR-05, Bro-10 and Bro-11) and their respective F_1_ hybrids (NKR-06 and Bro-12) were used in this study. The F_1_ hybrid NKR-06 was generated by crossing NKR-04 (maternal line) with NKR-05 (paternal line). The F_1_ hybrid Bro-12 was generated by crossing Bro-10 (maternal line) with Bro-11 (paternal line). All of the parental materials were self-bred at least six generations to harvest homozygous seeds. All of the F_1_ hybrids exhibited remarkable curd yield heterosis. The seeds of F_1_ hybrid broccoli and their parental lines (stored in Tianjin Kernel Vegetable Research Institute, Tianjin, China) were planted in a seeding room under the controlled conditions with a 16/8 h light/dark cycle at 25 °C and 22 °C, respectively. The 25-day-old seedlings were transplanted in the soil of the greenhouse with natural light cycle at 15 °C–28 °C. At 70 d after transplanting, the leaves, curds, and roots of each material (at least 20 individual plants) were measured and then harvested, immediately frozen in liquid nitrogen, and stored at − 80 °C for subsequent use in RNA and DNA isolation.

### RNA library construction and high-throughput sequencing

Total RNAs from the70-day-old curds of hybrid broccoli (NKR-06 and Bro-12) and their parents (NKR-04, NKR-05, Bro-10 and Bro-11) were isolated by TRIzol reagent (Invitrogen, CA, USA). RNAs with high purity (RNA integrity number, RIN > 8.0) were used to construct sequencing libraries by TruSeq Stranded mRNA LT Sample Prep Kit (Illumina, San Diego, CA, USA). These libraries were sequenced on the Illumina HiSeq™ 2500 sequencing platform and 150 bp/125 bp paired-end reads were generated. For each sample, at least 4 Gb raw data including over 50 million clean reads should be generated to get enough transcriptional information. In addition, to ensure the reliability of the sequencing data, three batches of independently isolated RNAs from a same sample were equally mixed to compose a RNA pool, which was used to construct sequencing library. In total, six RNA libraries were constructed and then sequenced with three technological replicates.

### Transcriptome data analysis

Clean reads were generated by discarding the low-quality reads (more than 20% bases in a read which have the quality score < 15), adaptors, reads with unknown base (N) content > 5% and other contaminants from the raw reads by NGS QC Toolkit software. All clean reads were annotated and classified by mapping them to the reference genome and transcript sequences of *Brassica oleracea var. oleracea* (https://www.ncbi.nlm.nih.gov/genome/10901?genome_assembly_id=59537) by bowtie2 and Tophat (http://tophat.cbcb.umd.edu/) with the default value. The expressed genes were confirmed based on the annotation information of the clean reads. The specific/common expression profiles of the genes among these samples were visualized by Venn diagram (http://bioinformatics.psb.ugent.be/webtools/Venn/). The expression level of each gene was calculated and normalized by the fragments per kilobase of transcript sequence per million base pairs sequenced (FPKM). Differential expression analysis of the genes was performed by using the R package DESeq (R version 3.0.1). The significantly DEGs between the two arbitrary samples were identified based on the following thresholds: |log_2_ (fold-change (A/B))| > 1 and corrected *p*-value < 0.05. A and B represent the normalized expression level of genes in any two samples, respectively. To explore the additive and non-additive expression patterns of genes, the gene expression levels in the F_1_ hybrids were compared with MPV. Non-additively expressed genes were identified based on the following thresholds: |log_2_ (normalized expression levels of F_1_ hybird/MPV)| > 1 and corrected p-value < 0.05. A hierarchical cluster analysis of the DEGs was conducted by the R package “gplots” (R version 3.0.1). The Pearson correlation coefficient and hierarchical cluster of all samples were analyzed by the “cor” and “hclust” in the R project (R version 3.0.1) with the default value. In addition, GO analysis of the DEGs were performed by using the agriGO (http:// bioinfo.cau.edu.cn/agriGO/) platform, and hypergeometric test was conducted to identify the significantly enriched GO terms (corrected p-value < 0.05). To further visualize the statistically overrepresented GO terms, the GO terms were analyzed by ReviGO (http://revigo.irb.hr/).

### qRT-PCR assay

The differential expression patterns of the genes detected by transcriptome data were validated by qRT-PCR analysis. The specific primer pairs were designed for the detection of corresponding genes (Additional file 1: Table S7). The *Actin* gene from broccoli was selected as internal control. Faststart Universal SYBR Green Master (Roche, Germany) was used in all experiments. The relative expression levels of the genes were calculated by the comparative 2^−ΔΔCT^ method according to the manufacturer’s recommendations. Three batches of independently isolated RNAs from each sample were used, and three technological replicates were performed to ensure the reliability of quantitative analysis.

### MethylRAD library construction and high-throughput sequencing

The 70-day-old curds of hybrid broccoli and their parents were used to isolate the genomic DNAs by cetyltrimethyl ammonium bromide (CTAB) method, respectively. 150–200 ng of DNAs were used to construct the MethylRAD library with slight modifications [50]. Briefly, the total DNAs were digested by using the methylation-dependent restriction enzyme FspEI (NEB, USA). FspEI can recognize 5-methylcytosine (5-mC) and 5-hydroxymethylcytosine (5-hmC) in the C^m^CGG and ^m^CHG sites, and generate a double-stranded DNA break on the 30 side of the modified cytosine at a fixed distance (N_12_/N_16_). Accordingly, symmetrical DNA methylated sites were bidirectionally cleaved by FspEI to generate 32-base-long fragments. Then, two adaptors were added to the digested DNAs by T4 DNA ligase (NEB, USA), and the ligation products were amplified by specific primers. The target DNA fragments were further purified and subjected to sequencing by Illumina HiSeq X-Ten sequencing platform [50]. Three batches of independently isolated DNAs from a same sample were equally mixed to compose a DNA pool, which was used to construct DNA methylation sequencing library. In total, six libraries were constructed and then sequenced with three technological replicates.

### DNA methylation data analysis

Adaptors and low-quality reads were discarded by NGS QC Toolkit software. The clean reads that did not contain the expected FspEI restriction site were further excluded, and the reads containing the methylated CG or CHG sites, named MethylRAD-tags, were identified. The MethylRAD-tags were mapped to the reference genome of *Brassica oleracea var. oleracea* by SOAP program (version 2.21) with two mismatches allowed. The distribution and density of methylated cytosine sites on chromosomes were calculated and visualized by the Integrative Genomics Viewer (IGV) [51]. Furthermore, the distributions of the methylated cytosine sites on different elements of the genome, especially on the different regions of genes, were evaluated. The DNA methylation levels of the sites were determined by the FPKM value, and the DNA methylation levels of the genes were then evaluated by summing the methylation levels of sites that were localized in the gene regions. The differential DNA methylation levels of sites and genes were identified by using the R package DESeq (R version 3.0.1). The thresholds were |log_2_ (fold-change (C/D)) > 1| and corrected *p*-value < 0.05. C and D represented the FPKM of sites or genes in any two samples, respectively.

## Additional files

Additional file 1: **Table S1.** View of the transcriptome data in two broccoli hybrid combinations. **Table S2.** Normalized expression levels and functional annotation of genes detected in two broccoli hybrid combinations. **Table S3.** DNA methylation ratio in two hybrid broccoli and their parents. **Table S4.** Relative DNA methylation levels at different regions of genes. **Table S5.** Distributions of CG and CHG sites with differential methylation levels. **Table S6.** Genes simultaneously showing differential methylation levels and differential expression levels. **Table S7.** primers used in the study. (ZIP 1392 kb)

Additional file 2: **Data set 1.** DEGs detected by pairwise comparison in NKR-06 and two parents. **Data set 2.** DEGs detected by pairwise comparison in Bro-12 and two parents. **Data set 3.** DEGs in the hybrids compared with both parents. **Data set 4.** Non-additively expressed genes in two hybrid triads. **Data set 5.** GO functional annotation of the DEGs in the NKR-04/− 05/− 06 hybrid triad. **Data set 6.** GO functional annotation of the DEGs in the Bro-10/− 11/− 12 hybrid triad. **Data set 7.** DNA methylation of CG and CHG sites detected in the hybrid broccoli and their parents. **Data set 8.** Differential DNA methylation levels of CG and CHG sites in NKR-06 hybrid triad. **Data set 9.** Genes with different methylation levels in NKR-06 hybrid triad. **Data set 10** Differential DNA methylation levels of CG and CHG sites in Bro-12 hybrid triad. **Data set 11.** Genes with differential methylation levels in Bro-12 hybrid triad. (ZIP 5944 kb)

Additional file 3: **Figure S1.** Expression levels of genes involved in several overrepresented biological processes. **Figure S2.** Expression profiles of several genes in BNR-H broccoli hybrid triad detected by qRT-PCR. **Figure S3.** Distributions of CG and CHG methylation sites at the different regions of genomes in the hybrids and their parents. **Figure S4.** DNA methylation levels at CHG sites in different regions of genes. (ZIP 34711 kb)

## Abbreviations

*ABCG26*: ABC transporter G family member 26
CTAB: Cetyltrimethyl ammonium bromide
DEGs: Differentially expressed genes
FPKM: Fragments per kilobase of transcript sequence per million base pairs
GO: Gene ontology
MPV: Mid-parent value
qRT-PCR: Quantitative real-time RT-PCR
QTL: Quantitative trait locus
TEK: Transposable element silencing via AT-hook
TSS: Transcription start sites
TTS: Transcription termination site
