# Supplementary material for: Transcriptome and DNA methylome reveal insights into yield heterosis in the curds of broccoli (Brassica oleracea L var. italic)
Source: BMC Plant Biol. 2018 Aug 13;18:168. doi: 10.1186/s12870-018-1384-4 (PMC6090608; doi:10.1186/s12870-018-1384-4)
Supplement: Supplementary file 1 — Table S1. View of the transcriptome data in two broccoli hybrid combinations. Table S2. Normalized expression levels and functional annotation of genes detected in two broccoli hybrid combinations. Table S3. DNA methylation ratio in two hybrid broccoli and their parents. Table S4. Relative DNA methylation levels at different regions of genes. Table S5. Distributions of CG and CHG sites with differential methylation levels. Table S6. Genes simultaneously showing differential methylation levels and differential expression levels. Table S7. primers used in the study. (ZIP 1392 kb) [file 12870_2018_1384_MOESM1_ESM.zip › 12870_2018_1384_MOESM1_ESM.pdf]

Transcriptome and DNA methylome reveal insights into yield heterosis in the curds of broccoli (*Brassica oleracea* L var. *italic*)

Hui Li [2], Jiye Yuan[1], Mei Wu[1], Zhanpin Han[2], Lihong Li[1], Hanmin Jiang[3], Yinglan Jia[1], Xue Han[1], Min Liu[4], Deling Sun[3], Chengbin Chen[1], Wenqin Song[1], Chunguo Wang[1**]

[1]College of Life Sciences, Nankai University, Tianjin, China
[2]College of Horticulture and Landscape, Tianjin Agricultural University, Tianjin, China
[3]Tianjin Kernel Vegetable Research Institute, Tianjin, China
[4]College of Life Sciences, Shandong Normal University, Jinan, Shandong, China
**Correspondence: email: wangcg@nankai.edu.cn

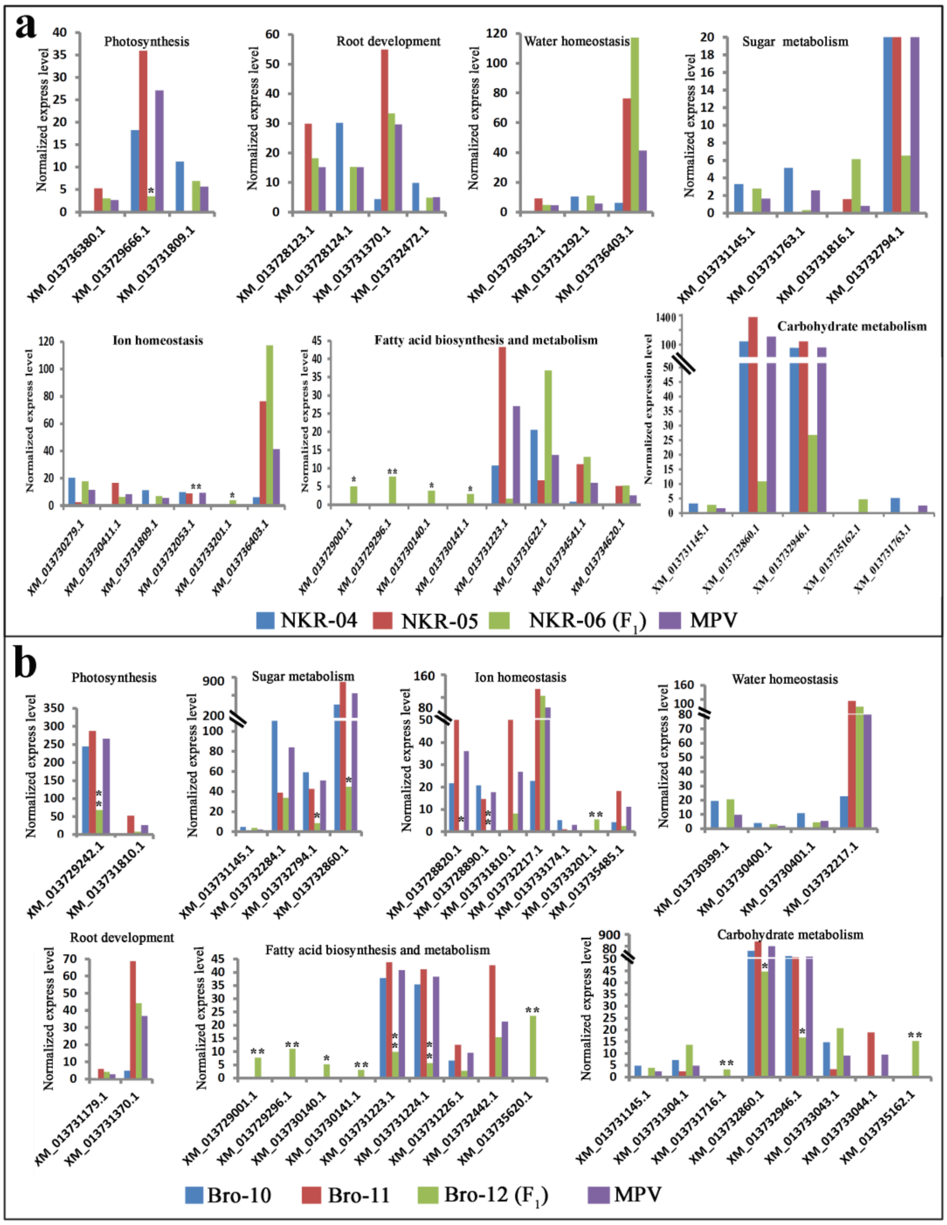

Figure S1 Expression levels of genes involved in several overrepresented biological processes in NKR-04/-05/-06 hybrid triad (a) and Bro-10/-11/-12 hybrid triad (b), respectively. * indicated the relative expression levels of the genes in the hybrid were different from the MPV (p-value < 0.05). ** indicated the relative expression levels of the genes in the hybrid were significantly different from the MPV (corrected p-value < 0.05). The functional annotations of these genes were showed in Additional file 1: Table S2.

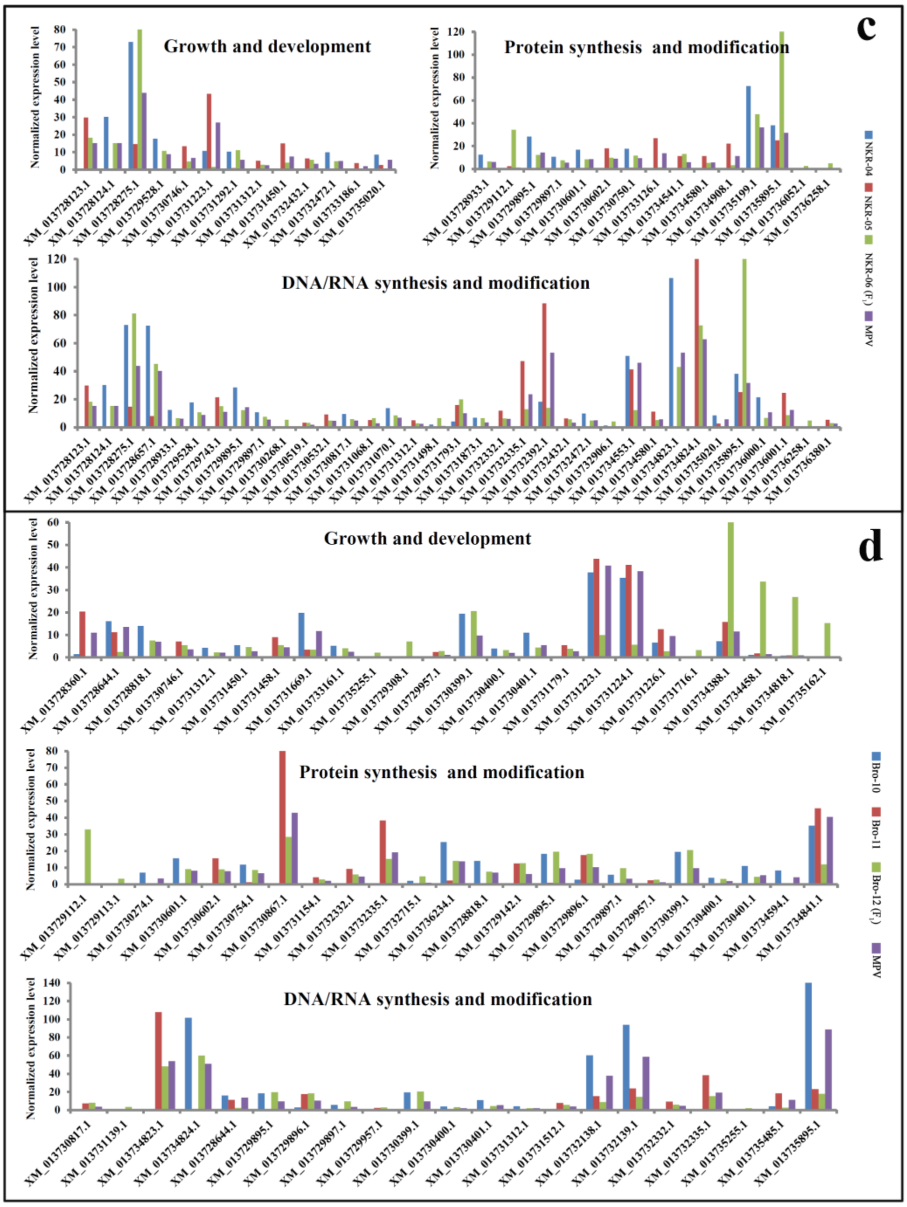

Figure S1 (**continued**) Expression levels of genes closely involved in several overrepresented biological processes in NKR-04/-05/-06 hybrid triad (c) and Bro-10/-11/-12 hybrid triad (d). The functional annotations of these genes were showed in Additional file 1: Table S2.

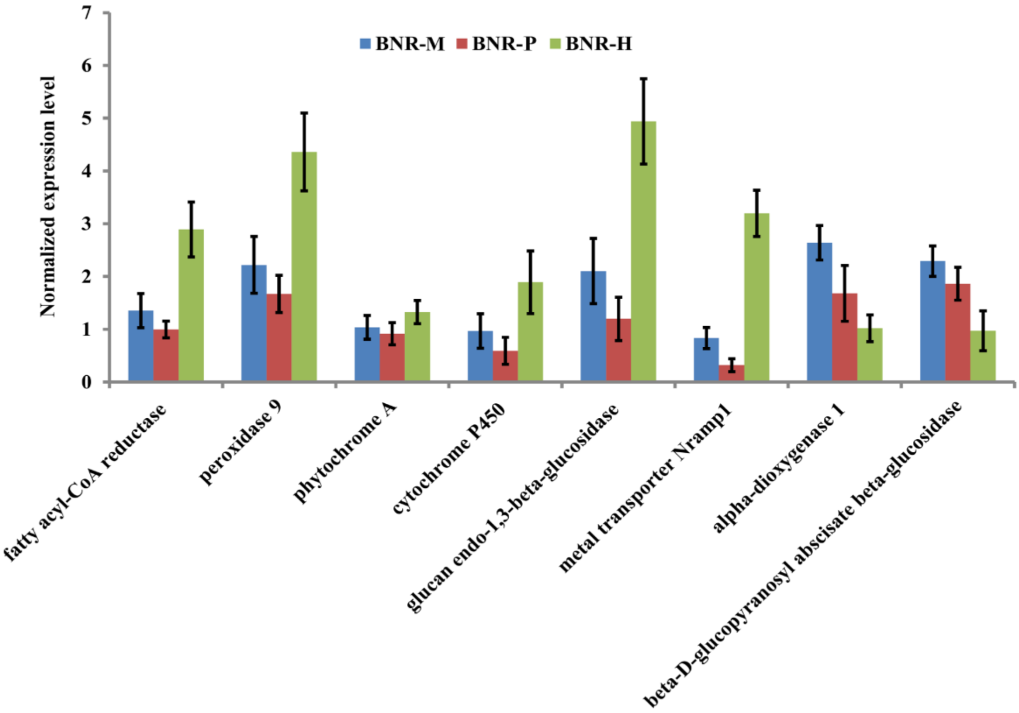

Figure S2 Expression profiles of several genes in BNR-H broccoli hybrid triad detected by qRT-PCR. All the selected genes showed significantly differentially expressed patterns in NKR-04/-05/-06 and Bro-10/-11/-12 hybrid triads. BNR-H is the F_1 hybrids of BNR-M (maternal line) and BNR-P (parental line).

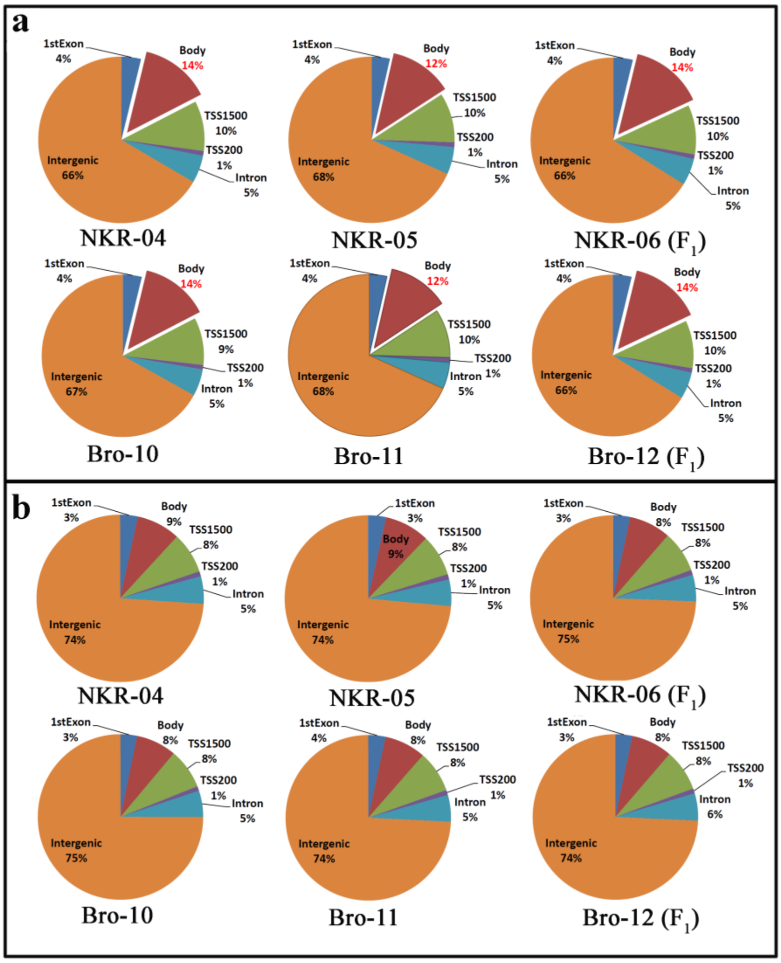

Figure S3 Distributions of CG (a) and CHG (H= T or A) (b) methylation sites at different regions of genomes in the hybrids and their parents. 1^{st}exon, Body, TSS1500, TSS200, intron and intergenic indicated the DNA methylation ratio at the first exon, gene coding regions, upstream 1500 bp of transcription start sites (TSS), upstream 200 bp of TSS, introns and intergenic regions, respectively.

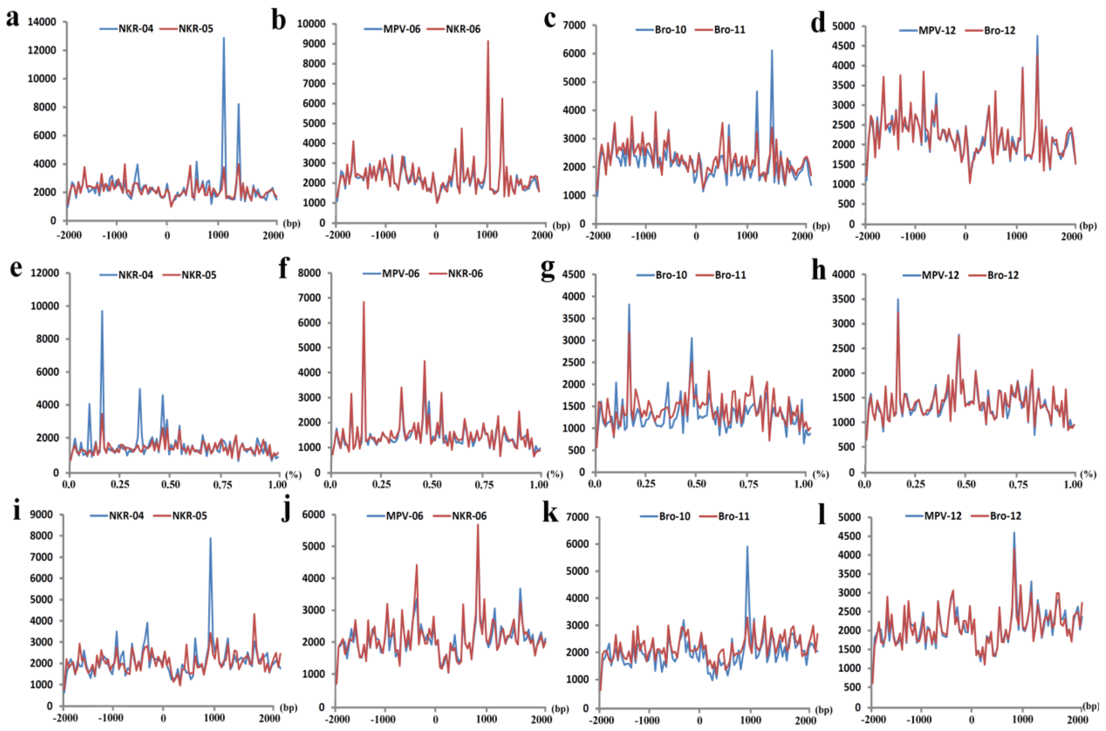

Figure S4 DNA methylation levels at the CHG (H =T or A) sites in different regions of genes. a, b, c and d indicated the relative DNA methylation levels at CHG sites of transcription start site (TSS) regions. e, f, g and h indicated the relative DNA methylation levels at the CHG sites of gene coding regions. i, j, k and l indicated the relative DNA methylation levels at the CHG sites of transcription termination site (TTS) regions. *X* axis showed the ± 2000 bp of TSS (a, b, c and d), the relative position of gene body (e, f, g and h) and the ± 2000 bp of TTS (i, j, k and l), respectively. *Y* axis showed the relative DNA methylation level. MPV-06 and MPV-12 indicated the mid-parent value of relative DNA methylation level in the NKR-04/-05/-06 hybrid triad and the Bro-10/-11/-12 hybrid triad, respectively.
