# Supplementary material for: Transcriptome and DNA methylome reveal insights into yield heterosis in the curds of broccoli (Brassica oleracea L var. italic)
Source: BMC Plant Biol. 2018 Aug 13;18:168. doi: 10.1186/s12870-018-1384-4 (PMC6090608; doi:10.1186/s12870-018-1384-4)
Supplement: Supplementary file 3 — Figure S1. Expression levels of genes involved in several overrepresented biological processes. Figure S2. Expression profiles of several genes in BNR-H broccoli hybrid triad detected by qRT-PCR. Figure S3. Distributions of CG and CHG methylation sites at the different regions of genomes in the hybrids and their parents. Figure S4. DNA methylation levels at CHG sites in different regions of genes. (ZIP 34711 kb) [file 12870_2018_1384_MOESM3_ESM.zip › Table S1.docx]

Transcriptome and DNA methylome reveal insights into yield heterosis in the curds of broccoli (*Brassica oleracea* L var. *italic*)

Hui Li^2^, Jiye Yuan^1^, Mei Wu^1^, Zhanpin Han^2^, Lihong Li^1^, Hanmin Jiang^3^, Yinglan Jia^1^, Xue Han^1^, Min Liu^4^, Deling Sun^3^, Chengbin Chen^1^, Wenqin Song^1^, Chunguo Wang^1**^

^1^College of Life Sciences, Nankai University, Tianjin, China

^2^College of Horticulture and Landscape, Tianjin Agricultural University, Tianjin, China

^3^Tianjin Kernel Vegetable Research Institute, Tianjin, China

^4^College of Life Sciences, Shandong Normal University, Jinan, Shandong, China

**Correspondence: email: [wangcg@nankai.edu.cn](mailto:wangcg@nankai.edu.cn)

**Table S1** View of the transcriptome data in two broccoli hybrid combinations.

| Sample | Bro-10 | Bro-11 | Bro-12 (F_1_) | NKR-04 | NKR-05 | NKR-06 (F_1_) |
| --- | --- | --- | --- | --- | --- | --- |
| Raw reads | 56096014 | 63459714 | 56633680 | 53095312 | 58844930 | 56752946 |
| Clean reads | 54453788 | 61788574 | 55137362 | 51568928 | 57075124 | 55180660 |
| Total mapped reads ^a^ | 43768582  (80.38%) | 49457491  (80.04%) | 44129040  (80.03%) | 41141572  (79.78%) | 45087981  (79.00%) | 44029598  (79.79%) |
| Multiple mapped reads ^a^ | 2035722  (3.74%) | 2362498  (3.82%) | 2015909  (3.66%) | 1960023  (3.80%) | 1995161  (3.50%) | 2059447  (3.73%) |
| Uniquely mapped reads ^a^ | 41732860  (76.64%) | 47094993  (76.22%) | 42113131  (76.38%) | 39181549  (75.98%) | 43092820  (75.50%) | 41970151  (76.06%) |
| Total mapped reads ^b^ | 50221223  (92.22%) | 56845920  (92.00%) | 49805671  (90.33%) | 46915973  (90.97%) | 52109056  (91.29%) | 49921915  (90.46%) |
| Multiple mapped reads^b^ | 22050442  (40.49%) | 25370304  (41.05%) | 22029424  (39.95%) | 20862707  (40.45%) | 22886090  (40.09%) | 22003770  (39.87%) |
| Uniquely mapped reads ^b^ | 28170781  (51.73%) | 31475616  (50.94%) | 27776247  (50.37%) | 26053266  (50.52%) | 29222966  (51.20%) | 27918145  (50.59%) |
| Identified genes | 45093  (92.89%) | 45529  (93.79%) | 46810  (96.43%) | 44040  (91.82%) | 44812  (93.43%) | 46442  (96.83%) |

Notice: a indicated that the clean reads were mapped to the *Brassica oleracea* var. *oleracea* genome. b indicated that the clean reads were mapped to the transcriptome of *Brassica oleracea* var. *oleracea.*
