# Supplementary material for: Transcriptome and DNA methylome reveal insights into yield heterosis in the curds of broccoli (Brassica oleracea L var. italic)
Source: BMC Plant Biol. 2018 Aug 13;18:168. doi: 10.1186/s12870-018-1384-4 (PMC6090608; doi:10.1186/s12870-018-1384-4)
Supplement: Supplementary file 3 — Figure S1. Expression levels of genes involved in several overrepresented biological processes. Figure S2. Expression profiles of several genes in BNR-H broccoli hybrid triad detected by qRT-PCR. Figure S3. Distributions of CG and CHG methylation sites at the different regions of genomes in the hybrids and their parents. Figure S4. DNA methylation levels at CHG sites in different regions of genes. (ZIP 34711 kb) [file 12870_2018_1384_MOESM3_ESM.zip › Table S7.docx]

Transcriptome and DNA methylome reveal insights into yield heterosis in the curds of broccoli (*Brassica oleracea* L var. *italic*)

Hui Li^2^, Jiye Yuan^1^, Mei Wu^1^, Zhanpin Han^2^, Lihong Li^1^, Hanmin Jiang^3^, Yinglan Jia^1^, Xue Han^1^, Min Liu^4^, Deling Sun^3^, Chengbin Chen^1^, Wenqin Song^1^, Chunguo Wang^1**^

^1^College of Life Sciences, Nankai University, Tianjin, China

^2^College of Horticulture and Landscape, Tianjin Agricultural University, Tianjin, China

^3^Tianjin Kernel Vegetable Research Institute, Tianjin, China

^4^College of Life Sciences, Shandong Normal University, Jinan, Shandong, China

**Correspondence: email: [wangcg@nankai.edu.cn](mailto:wangcg@nankai.edu.cn)

**Table S7** primers used in the study.

| Genes | Sequences |
| --- | --- |
| beta-1,3-galactosyltransferase 8  (XM_013729112.1) | XM_013729112-S: 5’ ATCAAGGCCGAGGGTTTACA3’ |
|  | XM_013729112-A: 5’ CATCCACGCTCCTAACGACA3’ |
| metal transporter Nramp1  (XM_013733201.1) | XM_013733201-S: 5’ GAAAGCGGACTGGCTCTAATG3’ |
|  | XM_013733201-A: 5’ CAAGAAGTGCGATGGCAAATA3’ |
| glucan endo-1,3-beta-glucosidase  (XM_013735162.1) | XM_013735162-S: 5’ CCTCTGCCTAAACCGACGAATA3’ |
|  | XM_013735162-A: 5’ GCGTAGCTTGCGTGCCAATAAA3’ |
| histidine-rich glycoprotein  (XM_013735463.1) | XM_013735463-S: 5’ CACCTTCTCCTCCCATCTTTCC3’ |
|  | XM_013735463-A: 5’ CGACGGTGGTTATGGTTGTTGT3’ |
| cytochrome P450  (XM_013729001.1) | P450-S: 5’ CTCTATGAACAATGTGACCAGGATG3’ |
|  | P450-A: 5’ CACGTCCCTCATTTCTTTCTCG3’ |
| ubiquitin receptor RAD23d  (XM_013754283.1) | XM_013754283-S: 5’ GAGGATTTGGTTGCTGATGTGA3’ |
|  | XM_013754283-A: 5’ GTTGAGGTAGACGTTGCGGTAG3’ |
| serine/threonine-protein kinase  CDL1(XM_013764210.1) | XM_013764210-S: 5’ GCTATTGTTCTTGATGTTTCGTTG3’ |
|  | XM_013764210-A: 5’ CCAAATCCCTCCTCAGCACC |
| fatty acyl-CoA reductase  (XM_013729296.1) | XM_013729296-S: 5’ CTATGGACGCTGAGACATTGGT3’ |
|  | XM_013729296-A: 5’ TGATTAGAAACGTCTTCCCTTGG3’ |
| alpha-dioxygenase 1  (XM_013731223.1) | XM_013731223-S: 5’TCCAAGACCAAGACGGTTTAGC3’ |
|  | XM_013731223-A: 5’TTTGTTCGCATCCCAGCAAGTA3’ |
| MYB35  (XM_013749968.1) | XM_013749968-S: 5’ CAAAGAATCCCTGGACAGATGAAG3’ |
|  | XM_013749968-A: 5’ TGTCTTTCCTGGTAGATGTTGTGC3’ |
| ARI15  (XM_013771788.1) | XM_013771788-S: 5’ ATCCGACAACACCAGCGAAGA3’ |
|  | XM_013771788-A: 5’ AAGCCCTTTCCTGTTTCCTCC3’ |
| beta-D-glucopyranosyl abscisate beta-  glucosidase (XM_013732860.1) | XM_013732860-S: 5’CTGTTAGGGTTAGTTCTGGTTCT3’ |
|  | XM_013732860-A: 5’TATGATTCTGACATCTATCTGGGTA3’ |
| RCC1 (XM_013729141.1) | XM_013729141-S: 5’ACCGATGTCAGGCTTGTTGCTT3’ |
|  | XM_013729141-A: 5’AGTCTTTCACAGAGGCGGGCTA3’ |
| cryptochrome-2 (XM_013730399.1) | XM_013730399-S: 5’GAAACTTGTGGAACGTGGGATC3’ |
|  | XM_013730399-A: 5’ATCAAACGCCAAGGAGGAGGAA3’ |
| phytochrome A  (XM_013748732.1) | XM_013748732-S: 5’CCAGCAATGTCAAAGCTAACCG3’ |
|  | XM_013748732-A: 5’TCCACATACTTCCCACCACTCG3’ |
| peroxidase 9  (XM_013734928.1) | XM_013734928-S: 5’CTGCTTCGTCCAGGGCTGTGAT3’ |
|  | XM_013734928-A: 5’GCTCCATTGAGGCTCGCTGTCG3’ |
| Actin | actin- S: 5’GCTCCTCTTAACCCAAAGGC3’ |
|  | actin- A: 5’CACACCATCACCAGAATCCAGC3’ |
